# Soft-robotic ciliated epidermis for reconfigurable coordinated fluid manipulation

**DOI:** 10.1126/sciadv.abq2345

**Published:** 2022-08-26

**Authors:** Ziyu Ren, Mingchao Zhang, Shanyuan Song, Zemin Liu, Chong Hong, Tianlu Wang, Xiaoguang Dong, Wenqi Hu, Metin Sitti

**Affiliations:** ^1^ Physical Intelligence Department, Max Planck Institute for Intelligent Systems, Stuttgart 70569, Germany.; ^2^ Department of Information Technology and Electrical Engineering, ETH Zurich, Zurich 8092, Switzerland.; ^3^ School of Medicine and College of Engineering, Koç University, Istanbul 34450, Turkey.

## Abstract

The fluid manipulation capabilities of current artificial cilia are severely handicapped by the inability to reconfigure near-surface flow on various static or dynamically deforming three-dimensional (3D) substrates. To overcome this challenge, we propose an electrically driven soft-robotic ciliated epidermis with multiple independently controlled polypyrrole bending actuators. The beating kinematics and the coordination of multiple actuators can be dynamically reconfigured to control the strength and direction of fluid transportation. We achieve fluid transportation along and perpendicular to the beating directions of the actuator arrays, and toward or away from the substrate. The ciliated epidermises are bendable and stretchable and can be deployed on various static or dynamically deforming 3D surfaces. They enable previously difficult to obtain fluid manipulation functionalities, such as transporting fluid in tubular structures or enhancing fluid transportation near dynamically bending and expanding surfaces.

## Teaser

A soft-robotic ciliated epidermis achieves reconfigurable coordinated fluid manipulation on various three-dimensional surfaces.

## INTRODUCTION

The reconfigurable coordination of a large number of cilia-like actuators that range from micrometer to millimeter scales in length on many animal epidermises endows them with the capability to manipulate the surrounding flows dexterously. As typical examples, starfish larvae (*1*), stentors (*2*), coral reefs (*3*), comb jellyfishes (*4*, *5*), and shrimps (*6*) can adaptively coordinate their numerous cilia, ctene plates, or swimmerets covered on their soft epidermises for locomotion, nutrient transportation, and predation. Similar reconfigurable coordination of multiple actuators also occurs inside the human body. Depending on the given physiological process, the cilia inside the fallopian tube (*7*) and ductus afferents (*8*, *9*) can dynamically adjust their rhythm and transportation direction to secure reliable delivery of eggs, sperms, and fertilized eggs to the targeted organs.

Inspired by the coordination of the cilia-like actuators for versatile flow control in nature, many artificial cilia have been proposed toward applications in microfluidic manipulation, biomedicine, and biomechanics. The artificial cilia made of hydrogels can be driven by pH change (*10*). However, this kind of cilia relies on the change of the environmental condition, limiting their application scenarios. The motions of the artificial cilia made of liquid crystal elastomers can be controlled by changing the wavelength, strength, and direction of the light (*11*, *12*). These light-driven cilia can be accessed individually and produce different collective behaviors. However, these cilia have not demonstrated their fluid manipulation capabilities, perhaps because of their limitations in actuation speed and amplitude. The requirements to the light sources make them only suitable to function in transparent environments. The artificial cilia can also produce in-phase beating excited by mechanical vibration (*13*) or acoustic wave (*14*), and remarkable flows at millimeters per second can be produced at the micrometer scale (*14*). However, these artificial cilia cannot produce out-of-phase collective coordination that may play an important role in fluid transportation in many biological systems (*15*, *16*), and their coordination cannot be reconfigured. The magnetically actuated artificial cilia, especially those made of magnetic soft composite materials, can realize superior single actuator kinematics and coordination among actuators (*15*, *17*, *18*). However, because of the difficulties of producing heterogeneous magnetic fields at high spatial resolution, it is challenging to let the magnetic artificial cilia function on arbitrary three-dimensional (3D) surfaces. In addition, since the magnetization profile of each actuator is preprogrammed, the cooperation of the actuators cannot be changed on the fly. The pneumatically driven cilia have demonstrated their ability to produce reconfigurable coordination since each cilium can be addressed individually (*19*, *20*). However, their internal cavity design and molding process make a single actuator longer than 1 cm. Artificial cilia driven by electrostatic force (*21*) and electrochemical reactions (*22*) have lengths less than 100 μm, and their coordination can be reconfigured to produce different flow patterns. However, they can only be fabricated on rigid silicon substrates, excluding them from being deployed on 3D surfaces.

To use the coordination of multiple actuators for flow manipulation on 3D surfaces at the millimeter scale, the cilia-inspired robotic system should have the following three key features. First, it should be able to achieve effective single actuator kinematics and the overall coordination of the multiple actuators for fluid transportation. Second, it should be able to function on various static and dynamically deforming 3D surfaces. Third, it should be able to reconfigure the coordination of the actuators on the fly for diverse fluid manipulation capabilities. Up to now, no artificial cilia can concurrently realize all these characteristics.

Here, we propose a soft-robotic ciliated epidermis that can propagate reconfigurable metachronal waves on static or dynamically deforming 3D surfaces. Each actuator of the ciliated epidermis is a millimeter-scale polypyrrole (PPy) bending polymer actuator controlled by electrical signals. By appropriately designing the positions of the electrodes, the beating of a single actuator or an actuator array can be individually controlled. By varying the beating kinematics of each actuator and the coordination among the actuators, the strength and direction of the flow can be controlled. Specifically, the fluids can be transported along the beating direction, perpendicular to the beating direction, or normal to the substrate surface. The soft-robotic ciliated epidermis can function on various 3D surfaces in static or dynamic conditions and alter the flow fields induced by dynamically deforming surfaces.

## RESULTS

### Design and fabrication of the ciliated epidermis

**Fig. 1.**
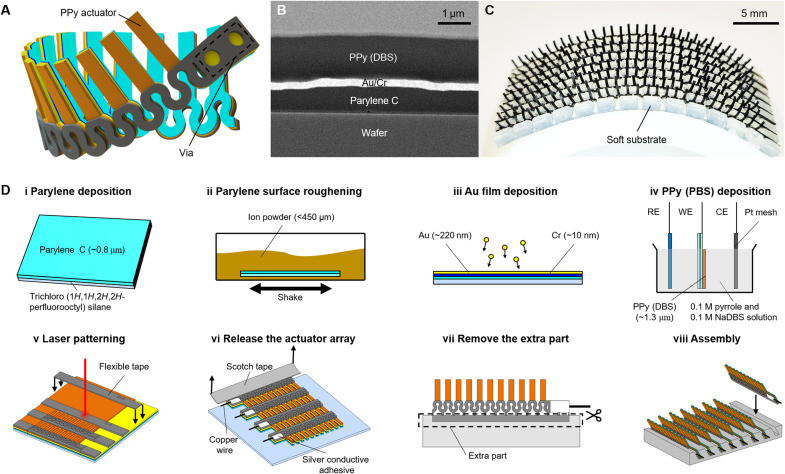
Design and fabrication of the soft-robotic ciliated epidermis. (**A**) Design of the ciliated actuator array. In the design shown here, all the actuators on the same array are actuated simultaneously. However, the actuators on the same array can also be independently controlled if the electrodes are separated. (**B**) Cross-sectional scanning electron microscopy image of the PPy actuator. The PPy layer is around 1.3 μm thick, the Au/Cr layer is around 230 nm thick, and the parylene C layer is around 0.8 μm thick. (**C**) Photo of a ciliated epidermis patched on a curved surface. (**D**) Schematic of a typical fabrication process of the ciliated epidermis.

The basic component of the soft-robotic ciliated epidermis is the ciliated PPy actuator array. A typical design of the actuator array is shown in Fig. 1A. The actuator array is composed of multiple layers (Fig. 1B). Multiple actuator arrays can be integrated to a soft substrate to produce the ciliated epidermis (Fig. 1C). The substrate layer of each actuator is a parylene C layer that has multiple functions. First, it acts as an insulation layer to prevent the direct contact between the electrodes of the adjacent actuators when they get entangled, which may cause the coupling of two actuators that are desired to be independently controlled. The entanglement of the actuators always happens when the actuators are densely packed. Second, it helps restrict the undesired deformation of the PPy layer. Without the restriction of the parylene C layer, the actuator may quickly curl toward the PPy side during the actuation and lose the ability to return to the flat state or even twist into a spiral (*23*), which has adverse effects on fluid manipulation performance. Third, it acts as a protection layer to minimize the damage to the actuators when peeling them away from the wafer (step vi in Fig. 1D). On top of the parylene C layer, a gold layer is sputtered. It acts as the actuator’s electrode and provides the electrical connection between the actuators on the same array. The gold layer has a rough surface (fig. S1) to facilitate its adhesion to the PPy layer (*24*). Note that the electrodes of the actuators on the same array can also be separated, as illustrated in fig. S2, if more controllable degrees of freedom (DOFs) are desired along the array direction (Materials and Methods, “Fabrication of the ciliated epidermis” section). On top of the gold layer, a PPy layer, doped with dodecylbenzenesulfonate (DBS), is deposited. The root of the actuator array is covered by a 120-μm-thick flexible insulation tape. It acts as the backbone of the whole actuator array for the ease of the assembly process (step viii in Fig. 1D). Vias are created on the tape layer to let the electricity connection reach the gold layer beneath. The actuator array has a serpentine shape to achieve flexibility and stretchability (*25*). The actuator arrays are finally planted into the sockets of the soft surfaces to create the soft-robotic ciliated epidermis that can be deployed to 3D surfaces (step viii in Fig. 1D).

### Characterization of a single ciliated actuator array

DBS^−^ is used to dope the PPy during electropolymerization, which can produce actuators with high conductivity and mechanical properties (*26*). DBS^−^ is a large anion that keeps immobile in the polymer matrix. When injecting electrons to the actuators (reduction state), small mobile cations and solvent will be drawn into the PPy to keep charge neutrality, causing it to expand. When extracting the electrons from the actuators (oxidation state), the small mobile cations will be expelled from the PPy, causing it to contract (*27*). If not explicitly stated otherwise, the electrolyte used is 0.1 M NaDBS solution, in which Na^+^ acts as the small mobile cation. The electrolyte has a similar viscosity as the water at room temperature (*28*).

**Fig. 2.**
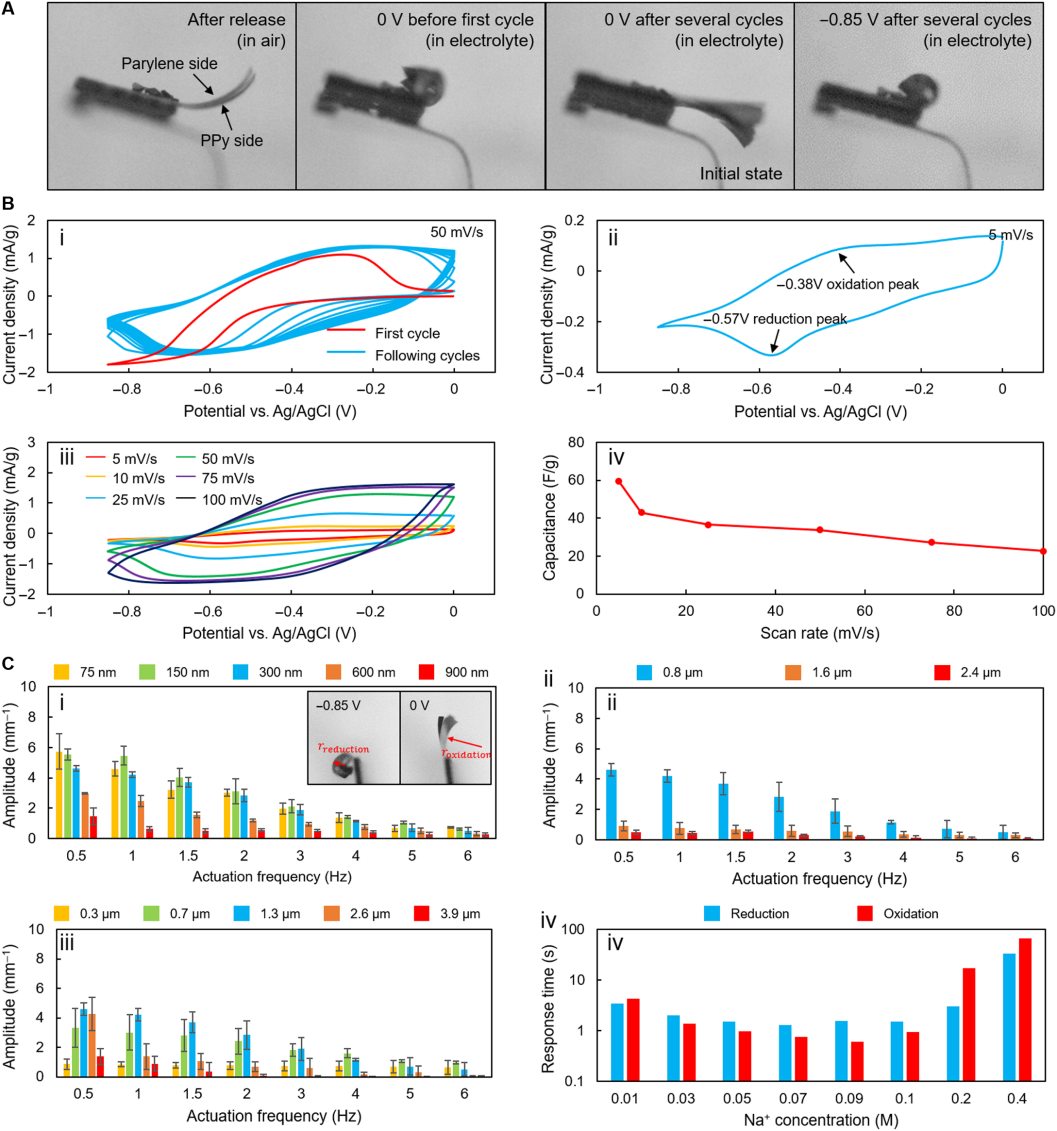
Characterization of PPy actuators. (**A**) The actuators slightly bend toward the side of the parylene C layer after being released from the wafer. When putting the actuators in the electrolyte, the actuators curl toward the side of the parylene C layer with a very large curvature. After actuating the actuators in the electrolyte for several cycles with a square wave (−0.85 to 0.1 V; duty cycle, 50%), the actuators gradually expand. The actuators return to the initial state at 0 V and achieve the maximum curvature at −0.85 V. (**B**) Cyclic voltammograms at different measurement conditions (i to iii) and the capacitance calculated from the *C*-*V* curve (iv). (**C**) Influence of different factors on the bending amplitude. (i) Influence of gold layer thickness. (ii) Influence of parylene C layer thickness. (iii) Influence of PPy layer thickness. (iv) Influence of Na^+^ concentration. In (i) to (iii), the values are averaged from the measurements of three samples.

The actuators need to be activated by applying several reduction and oxidation cycles before obtaining a stable beating performance. When the free-standing actuators are immersed in the electrolyte, they immediately bend toward the side of the parylene C layer, forming a very large curvature (Fig. 2A). After applying a square signal ranging from −0.85 to 0.1 V (all the voltage values given here are relative to an Ag/AgCl reference electrode) for several cycles, the actuators gradually expand and are able to return to their initial states when the input voltage is kept at 0 V. At the initial state, the actuators are flat or bend a little toward the PPy side. The thicknesses of the actuators are not exactly identical because of the fabrication nonuniformities, resulting in differences in initial bending curvatures. The abnormal first redox cycle of the PPy, which causes the abnormal behaviors of the actuators in the first several actuation cycles, can be observed by conducting cyclic voltammetry, where the current-voltage (*C*-*V*) curve of the first cycle is notably different from the *C*-*V* curves from the following cycles (Fig. 2B, i). For this reason, a square control signal ranging from −0.85 to 0.1 V is applied to activate the actuators for several cycles before use.

The actuator can reach its maximum bending curvature when fully reduced and return to its initial state when fully oxidized. However, as the scan rate of the control signal increases, the PPy becomes harder and harder to complete an entire redox cycle, as visualized in Fig. 2B (ii and iii), where the oxidation and the reduction peaks of the *C*-*V* curves gradually separate with the increasing scan rate. Moreover, the amount of the cations coming into the PPy also drops with the increasing scan rate (Fig. 2B, iv), which explains why the actuator amplitude decreases as the actuation frequency increases (Fig. 2C).

The geometric design of the actuators, especially the thickness of each layer, can greatly influence its bending amplitude. The bending amplitudes of the actuators with different thickness designs are characterized under a square waveform ranging from −0.85 to 0 V with frequency ranging from 0.5 to 6 Hz (Fig. 2C). When the actuator is fully reduced, the curvature is measured as 1/*r*_reduction_. When the actuator is fully oxidized, the curvature is measured as 1/*r*_reduction_. If the center of the fitted circle locates at the parylene side (left side), the curvature is counted as a positive; otherwise, it is counted as a negative. The bending amplitude is then defined as 1/*r*_reduction_ − 1/*r*_reduction_.

Increasing the gold layer thickness can improve the conductivity of the electrode, which is favorable for improving the response speed of the actuator (*29*). However, an overly thick gold layer (>300 nm) notably decreases the actuators’ bending amplitude (Fig. 2C, i). Similar results can also be observed in actuators with varying parylene C layer thickness (Fig. 2C, ii). Although a thin parylene C layer can lead to a larger bending amplitude, the actuators with an overly thin parylene C layer are always broken or have inhomogeneous shapes after being released from the wafer (fig. S3A). The thickness of the PPy layer influences both the bending amplitudes and the frequency response of the actuators (Fig. 2C, iii). A thin PPy layer can help to increase the maximum actuation frequency of the actuator because it takes the small cations less time to diffuse into the PPy layer (*29*). However, an overly thin PPy layer may lead to a decrease in bending amplitude. In our experiments, the actuators with PPy layers thinner than 1.3 μm (0.3 and 0.7 μm) have a better frequency response (the beating amplitude decreases more slowly with the increasing frequency). However, their beating amplitudes are smaller than the actuators with 1.3-μm PPy layer at low frequencies (<4 Hz). Actuators with PPy layers thicker than 1.3 μm (2.6 and 3.9 μm) perform poorly in both beating amplitude and frequency response. In addition to the thickness of each layer, other geometric parameters, including the length and width of an actuator, are also investigated (fig. S4). These parameters have less influence on actuator’s bending amplitude and frequency response.

The relation between the thickness of each layer and the bending curvature is nonlinear and can be described by Timoshenko’s bi-strip bending model (*30*). However, maximizing the bending curvature is not the only optimization target when determining the thickness of each layer. First, the actuator that bends into a circle (as shown in Fig. 2A) actually loses the capability to transport fluids, although its curvature is very large. Second, the frequency response is a very important factor that influences the fluid transportation performance. The thinner the PPy layer, the higher the maximum actuation frequency. Third, the thickness of each layer also influences the fabrication quality and uniformity. Thicker layers can make the qualities of the actuators more uniform. Fourth, overly thin layers may make the actuators too brittle, inducing great challenges in assembly and deployment and resulting in low load capacity. In our study, the thicknesses of the parylene C layer, the gold layer, and the PPy layer are determined to be 0.8, 230, and 1.3 μm. These design parameters are trade-offs among producibility, frequency response, beating amplitude, and fluid manipulation performance.

In addition to different geometric design parameters, the concentration of the small cations also has a great impact on actuator’s response time. Since the volume change of PPy is incurred by the ion exchange, the actuators cannot function in deionized (DI) water. To determine the threshold of the cation concentration for maintaining the actuator’s function, we conduct experiments to measure the response time of the actuator in different Na^+^ concentrations (Fig. 2C, iv). We begin the experiments in DI water and gradually add high-concentration NaDBS solution into it to get electrolytes with different Na^+^ concentrations. At each concentration, we first apply a constant voltage of 0 V for more than 10 s to fully oxidize the actuators (when the actuators fully expand) and then apply a step signal to −0.85 V. The time required to fully reduce the actuators (when the actuators fully bend) is determined using a high-speed camera. The time required to fully oxidize the actuators is recorded in a similar way. When the Na^+^ concentration is low (0.01 to 0.03 M/liter), the time required to fully oxidize or reduce the actuators is longer than 0.1-M Na^+^ solution. When the Na^+^ concentration reached 0.05 to 0.09 M, the response time of the actuator becomes comparable to 0.1 M Na^+^ solution. When Na^+^ concentration increases to 0.2 M or higher, the response time of the actuators increases greatly. These results indicate that the ideal Na^+^ concentration range for PPy actuators is 0.05 to 0.1 M.

### Two beating modes for fluid transport

**Fig. 3.**
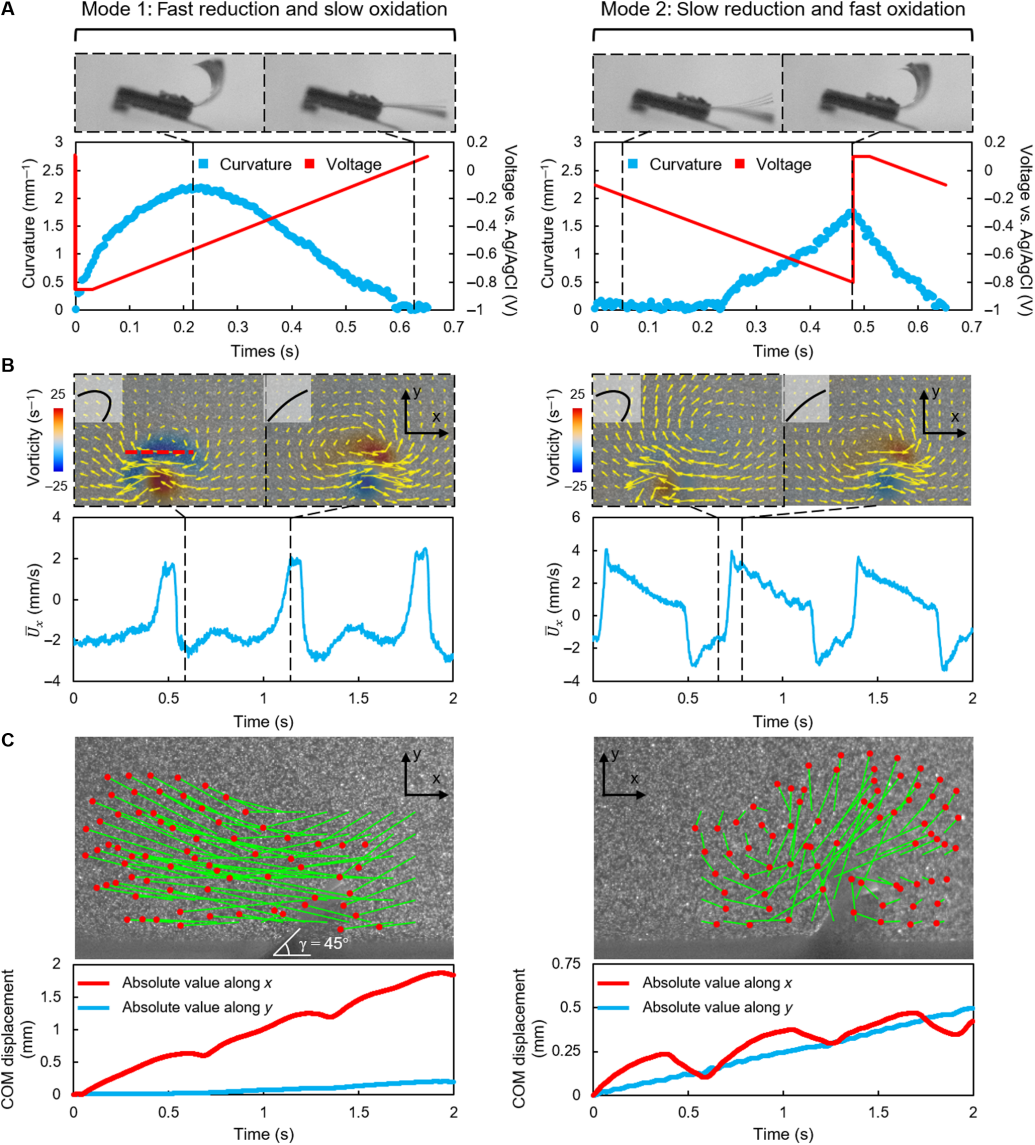
Performance characterization of a ciliated actuator array. (**A**) Actuator kinematics quantified by curvature variation. Two control signals are prescribed to produce different oxidation and reduction durations. (**B**) Flow structures induced by the two beating modes. By tuning the duration of the oxidation and reduction phases, the fluids are transported in different directions. The flow velocities are averaged along the red dashed line. (**C**) Transportation performances of the two beating modes. Massless virtual particles are tracked on the basis of the flow fields obtained by particle image velocimetry measurements. The center of mass (COM) displacements parallel and normal to the substrate are plotted. The red dots indicate the final positions of the virtual particles after three beating cycles. The green lines connect the initial and final positions of the particles. In (B) and (C), the planting angle γ of the actuator is 45°. The actuators shown here are 1.5 mm in length.

By controlling the sweeping speed of the control signal, the bending amplitude and the reduction/oxidation duration of the actuators can be controlled (fig. S5). Following this strategy, we design two control signals that can produce two beating modes with different oxidation and reduction duration ratios while still achieving acceptable bending amplitudes and frequencies (Fig. 3A). In beating mode 1, the control signal markedly drops from 0.1 to −0.85 V in 1 ms, then keeps the voltage at −0.85 V for 311 ms, and gradually recovers to 0.1 V in 652 ms in the last stage. This control signal can induce a maximum bending curvature of 2.2 mm^−1^ and a faster bending speed toward the parylene C layer than toward the PPy layer. The time spent to reach the maximum curvature from the initial state takes up 32.5% of one complete actuation cycle (0.652 s). In beating mode 2, the control signal gradually drops from 0.1 to −0.85 V in 652 ms, then suddenly returns to 0.1 V in 1 ms, and finally keeps at 0.1 V for 311 ms. This control signal can induce a maximum bending curvature of 1.75 mm^−1^ and a faster bending speed toward the PPy layer than toward the gold layer. The time spent to reach the minimum curvature from the initial state takes up 27.6% of one complete actuation cycle (0.652 s).

Modes 1 and 2 can induce fluid flow being transported in different directions. In both modes 1 and 2, the strength of the average flows ($|{\bar{U}}_{x}|$ is calculated along the red dashed line shown in Fig. 3B; Materials and Methods, “Calculation of the average flow along a line and the planar flow rate Q” section) in the –*x* and +*x* directions peaks at the instants when the actuators reach the maximum and minimum curvatures, respectively. In mode 1, the actuator creates a larger peak $|{\bar{U}}_{x}|$ in the –*x* direction (3.07 mm/s) than in the +*x* direction (2.50 mm/s), resulting in a net flow in the –*x* direction. The time-averaged flow speed during one beating cycle is 1.38 mm/s. In mode 2, the peak $|{\bar{U}}_{x}|$ in the +*x* direction is notably increased (4.06 mm/s) because of the faster oxidation speed, resulting in a net flow in the +*x* direction. The time-averaged flow speed during one beating cycle is 0.52 mm/s. For the actuators that are 1.5 mm in length as used here, the maximum Reynolds number estimated on the basis of the peak velocity of the actuator tip is around 30, indicating that the actuators work within an intermediate Reynolds number regime, where inertial fluidic forces are important. To better visualize how the fluids around the actuators are influenced, we also track virtual massless particles in flow fields obtained by particle image velocimetry (PIV) experiments (Fig. 3C; Materials and Methods, “Virtual particle tracing based on PIV experiments” section). The displacement of the center of mass of the virtual particles induced by mode 2 is shorter than that induced by mode 1 within the same period of time, suggesting that mode 1 can transport fluids faster.

In addition to the control signal, the fluid transportation performance of the actuators is also influenced by other factors, such as the actuator length and the planting angle γ. The fluid transport by such reciprocal beating motion can only be achieved if the Reynolds number is high enough, according to the Purcell’s scallop theorem (*31*). In view of the fact that the response frequency of the actuator is capped by the ion diffusion speed, increasing the actuator length is the most practical way to escape from the Stokes flow regime. The average flow velocity increases rapidly as the actuator’s length increases, based on the simulation shown in fig. S6. However, long actuators are easy to get entangled during actuation (fig. S3B). To alleviate the entanglement of the adjacent actuators while still achieving effective fluid transport, the actuators used in this work have lengths between 1 and 2 mm. Besides, simply planting the actuators vertically (γ = 90°) can restrict the flow toward the +*x* direction, decreasing the fluid transport capability of mode 2. By tilting the actuators relative to the substrate (decreasing γ), the performance of mode 2 can be improved. However, an overly small planting angle can degrade the fluid transport performance of mode 1 (fig. S7). To balance the performances of modes 1 and 2, we keep the planting angle γ = 45° throughout the paper.

### Effect of the phase shift between different actuator arrays

Changing the phase shift between the actuator arrays provides an avenue to control the flow direction and strength along the actuator’s beating direction or along the direction normal to the substrate. For demonstration, a soft ciliated epidermis with eight rows of actuator arrays is fabricated and deployed on a flat substrate (movie S1). Each actuator array has 16 actuators with a length of 1.5 mm and a width of 0.3 mm. The distance between the adjacent arrays is 1.875 mm. This distance is determined to avoid mechanical interference while maximizing the flow interaction between the adjacent actuator arrays.

**Fig. 4.**
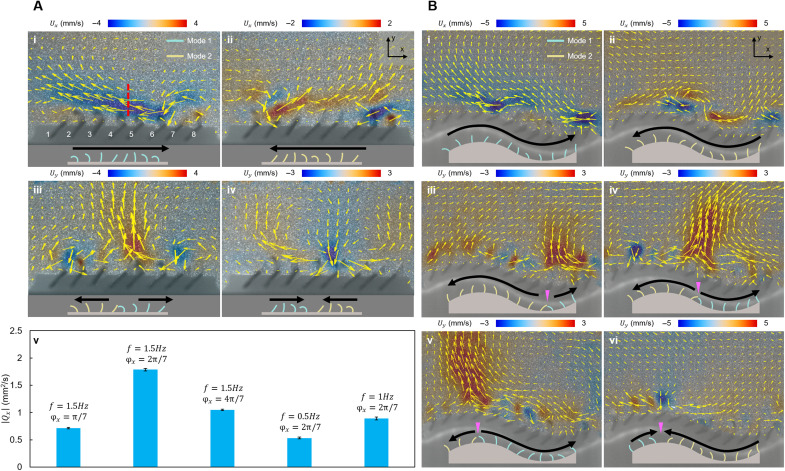
Reconfiguration of the beating mode and the phase shift between the actuator arrays for enabling different fluid manipulation performances. (**A**) Producing a phase shift between the actuator arrays on a flat surface. (i to ii) By tuning the directions of the metachronal waves and the beating modes of the actuators, the fluid can be transported parallel to the substrate in two directions. (iii to iv) By producing metachronal waves with different transmitting directions, the fluid can be pushed away or drawn toward the substrate. (v) By changing the phase shift (φ*_x_*) between the actuator arrays and the beating frequency of the actuators, the flow strength parallel to the substrate can be tuned, which is quantified by ∣*Q_x_*∣. *Q_x_* is calculated along the red dashed line in (i). The values are averaged from ∣*Q_x_*∣ of five consecutive beating cycles. (**B**) Producing phase shift between the actuator arrays on a sinusoidal substrate. (i to ii) By tuning the directions of the metachronal waves and the beating modes, the ciliated epidermis can transport fluids along the substrate in two directions. (iii to v) By tuning the reversal point of the two metachronal waves departing from each other, the locations where the fluids are pushed away from the substrate can be shifted. (vi) By reversing the directions of the metachronal waves in (v), the fluids can be drawn toward the substrate at the same location. In (A) and (B), the side views of the ciliated epidermis are depicted below the flow fields. The black arrows indicate the directions of the metachronal waves.

Keeping all actuators beating with mode 1, the ciliated epidermis can transport fluid to the –*x* direction by producing a metachronal wave that transmits from actuator array 1 to actuator array 8 (Fig. 4A, i). The direction of the metachronal wave is determined on the basis of the fact that the erected actuator can resist the fluid being transported. As shown in fig. S8, if the actuator located downstream lags behind the one upstream, the flow will be blocked by the erected downstream actuator, decreasing the maximum flow speed. Such antiplectic wave, i.e., the wave with propagating direction that is opposite to the flow direction, exists in many biological systems in a wide range of Reynolds number regimes, such as the cilia covered on Paramecium (*32*) and the swimming limbs of the mantis shrimps (*6*), and its contribution to fluid transportation has been validated (*15*, *16*). Changing the fluid transport direction can be realized by switching the beating mode to mode 2 and reversing the propagating direction of the metachronal wave (Fig. 4A, ii).

Inspired by the reversal of the surface beating direction of the cilia observed in starfish larvae (*1*), we can transport fluids along the direction normal to the substrate by simultaneously producing two metachronal waves in opposite directions. To push the fluids away from or draw the fluids toward the substrate, flows toward the middle position or toward two sides of the ciliated epidermis are produced by reconfiguring the beating kinematics of the actuator arrays. The corresponding beating modes and the metachronal wave directions are indicated in Fig. 4A (iii and iv). The maximum flows normal to the substrate are produced at the reversal point of the metachronal waves, just like that observed in the biological system (*1*).

The strength of the fluid transport by using a unidirectional metachronal wave, which is quantified by *Q_x_* (Materials and Methods, “Calculation of the average flow along a line and the planar flow rate *Q*” section) calculated along the red dashed line shown in Fig. 4A (i), can be controlled by changing the phase φ*_x_* between the adjacent actuator arrays (Fig. 4A, v). We set φ*_x_* to π/7, 2π/7, and 4π/7 and observe that the ciliated epidermis can produce the strongest transportation at φ*_x_* = 2π/7. We also prescribe two more actuation signals with a lower beating frequency while keeping the time duration ratio of each phase unchanged. These two signals result in a reduction of *Q_x_*.

The flexibility of the ciliated epidermis allows it to function on nonflat surfaces. The deformable ciliated epidermis with 12 actuator arrays is deployed on a sinusoidal surface (movie S2). By applying the beating modes and the metachronal waves shown in Fig. 4B, the fluids can be transported parallel or perpendicular to the local substrate. The reversal point can be shifted along the curved substrate in an agile manner, changing the location where the fluid is pushed away or drawn toward the surface. The variation of the surface curvature induces nonhomogeneous interarray distance, although the actuators are initially planted with equal spacing. This change in interarray distance can alter the flow manipulation performance locally. For example, the ciliated epidermis can produce a stronger flow along the *y* direction when the reversal point shifts to the concave location than to the convex location of the substrate (comparison between Fig. 4B, v and vi). At the convex location, the distance between the actuators on the adjacent rows is overly small, making the actuators easy to tangle together when beating reversely.

### Effect of the phase shift along the actuator array direction

The actuators sitting on the same actuator array can also be independently controlled by separating the electrodes of these actuators (fig. S2; Materials and Methods, “Fabrication of the ciliated epidermis” section). More controllable DOF along an array enables the epidermises to generate metachronal waves both along and perpendicular to the actuators’ beating direction. To investigate the influence of the metachronal wave along the actuator array direction, we develop a ciliated epidermis with 12 actuators patterned as a 3 × 4 matrix (movie S3). Each actuator is 1.5 mm in length and 0.3 mm in width. The distance between the adjacent actuators along an array is 1 mm, and the distance between the actuators on adjacent rows is 1.875 mm. The flow fields above the ciliated epidermis are studied.

**Fig. 5.**
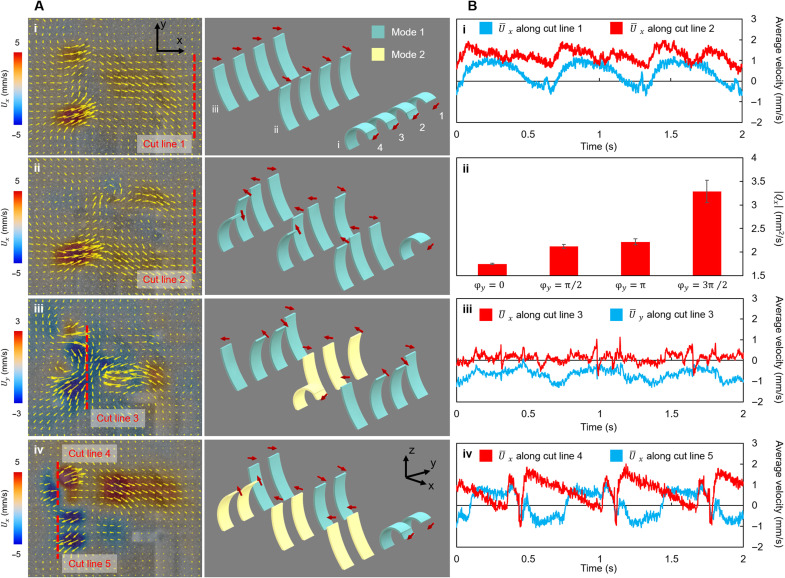
Reconfiguration of the beating mode and the phase shift along the array direction for enabling different fluid manipulation performances. (**A**) Producing phase shift between the actuator arrays on a flat substrate. (i to iv) Flow fields produced by the ciliated epidermis with out-of-phase motion along the array (φ*_y_* ≠ 0). The phase shift between the arrays (φ*_x_*) is kept to be 2π/7. (**B**) Quantification of the flow field. (i) Average flow velocities in the *x* direction (${\bar{U}}_{x}$) along cut lines 1 and 2. ${\bar{U}}_{x}$ achieved at φ*_y_* = 2π/7 is larger than at φ*_y_* = 0. (ii) *Q* calculated across cut line 2 at different φ*_y_*. The values are averaged from ∣*Q_x_*∣ of five consecutive beating cycles. (iii) ${\bar{U}}_{x}$ and ${\bar{U}}_{y}$ along cut line 3. The fluid transportation along the *x* direction is suppressed, while the transportation along the *y* direction is amplified. (iv) ${\bar{U}}_{x}$ along cut lines 4 and 5. Two fluid transportation regions with different transportation directions are achieved.

By only transmitting the metachronal wave from row i to row iii (φ*_x_* = 2π/7) with all actuators beating in mode 1, the fluids are transported along the +*x* direction (Fig. 5A, i). The average velocity, ${\bar{U}}_{x}$, measured along the cut line 1 in three actuation cycles shows a periodically pulsatile pattern with a peak-to-valley value of 1.94 mm/s (Fig. 5B, i). During the periods when all the actuators are recovering from the maximum curvature to the initial states, ${\bar{U}}_{x}$ even turns to be negative, suggesting that the fluids are transported backward. When a metachronal wave transmitting from column 4 to column 1 with φ*_y_* = 3π/2 is produced (Fig. 5A, ii), the ${\bar{U}}_{x}$ profile (calculated along cut line 2) is translated upward and compressed vertically (Fig. 5B, i). Specifically, the maximum and minimum values of the average velocity rise to 1.93 and 0.33 mm/s, respectively, and the peak-to-valley value drops to 1.63 mm/s, indicating that the instantaneous flow becomes stronger and more continuous. With the metachronal wave traveling along the array direction, there always exist actuators that are beating to accelerate the fluids, which may be the reason why the negative values disappear from the ${\bar{U}}_{x}$ profile. Producing metachronal waves along the array direction with other phase shifts is also observed to enhance the fluid transportation, as illustrated in Fig. 5B (ii). Such enhancement to fluid transportation by producing metachronal waves in the array direction is also observed in cilia arrays at low Reynolds numbers through numerical investigation (*33*).

The fluid can also be transported in the direction along the actuator array. As shown in Fig. 5A (iii), a net flow along the –*y* direction can be observed between row ii and row iii if all actuators along row ii beat with mode 2 and all actuators along row iii beat with mode 1. To generate such flow, the metachronal waves along the two actuator arrays transmit from column 4 to column 1 with a phase shift φ*_y_* = 3π/2, while the actuators at the same column beat in phase. The instantaneous average velocity ${\bar{U}}_{y}$ along the cut line 3 shows that the flow perpendicular to the beating direction can be continuously produced while the flow along the beating direction is impeded (Fig. 5B, iii). This observation suggests that the flow generated by a beating actuator should no longer be simplified as a 2D flow in this case, and the metachronal coordination of the actuators can amplify the flow perpendicular to the actuators’ beating direction.

The ability to independently control the actuators on the same actuator array also enables us to obtain flow fields with distinct flow properties in parallel. As shown in Fig. 5A (iv), the actuators on columns 1 and 2 all beat with mode 1, while the actuators on columns 3 and 4 all beat with mode 2. The instantaneous average velocities ${\bar{U}}_{x}$ measured along cut lines 4 and 5 show that the two groups of columns with different actuator beating kinematics produce two flow regions with different fluid transportation properties (Fig. 5B, iv).

### Ciliated epidermises on different 2D and 3D surfaces

**Fig. 6.**
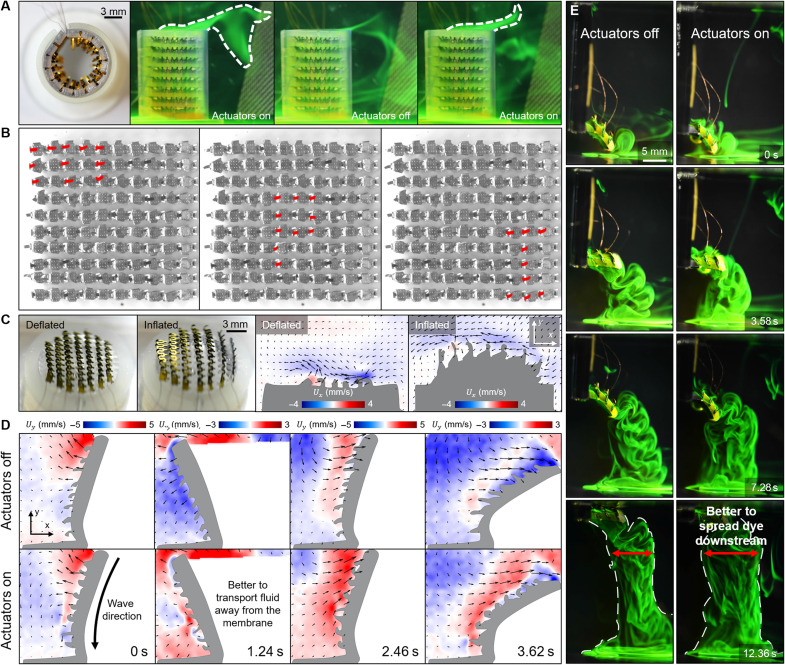
Deployment of the ciliated epidermis on different curved and dynamically deforming surfaces. (**A**) Deployment of the ciliated epidermis inside a cylindrical tube. The ciliated epidermis is wrapped and patched to the inner surface of the tube. When the actuators are turned on, the dyes injected into the tube are pumped out. (**B**) Independently controlling each actuator of a 10 × 10 actuator matrix on a flat surface. The actuator matrix can dynamically display different letters at different locations. The actuators being activated are shaded with a red color. (**C**) Deployment of the ciliated epidermis on a dynamically stretchable 3D surface. The ciliated epidermis is patched to the outer surface of a pneumatically driven soft actuator. The epidermis can still transport fluids near the substrate when the surface is stretched due to inflation. (**D**) Enhancing the downstream flow produced by a flapping membrane fixed at one end. The ciliated epidermis is patched to one side of the deforming membrane. When the ciliated epidermis is turned on, the flapping substrate produces a stronger flow downstream. (**E**) Changing the wake flow shedding pattern of a flapping membrane translating upward with a constant speed. The ciliated epidermis is patched on top of a flapping membrane. When the ciliated epidermis is turned on, more dyes are shed from the top layer of the membrane. The red double arrows indicate the change in dye trace area.

Fluid transport in confined environments through metachronal coordination is ubiquitous in biological systems (*8*, *34*, *35*). The deformability and stretchability of our soft-robotic ciliated epidermis and the decoupling between the actuation input and the surface morphology enable us to deploy it in tubular structures for fluid transportation. A ciliated epidermis, which has a size of 35.0 mm by 17.5 mm and is integrated with eight actuator arrays, is deployed in a hollow cylindrical tube with a height of 18 mm and an inner diameter of 10 mm (Fig. 6A). Each actuator is 2 mm in length and 0.35 mm in width. The distances between the adjacent actuator arrays and the actuators on the same array are 2 mm. During the experiment, fluorescein dye is injected into the cylindrical tube when the actuators are at rest. Since the dye is heavier than the electrolyte, it does not float upward from the tube. When the actuators beat with mode 1 and transmit a metachronal wave from top to bottom with φ = 2π/7, the dyes inside the tube are gradually pushed out. If the actuators are turned off again, the dyes will no longer come out from the tube (movie S4).

Increasing the number of independently controlled actuators may lead to a bulky control circuit, which is undesired when deploying the soft-robotic ciliated epidermis in autonomous platforms. To tackle this challenge, we propose a sequential scanning method for large-scale actuator matrix control. This method can be applied to a ciliated epidermis with *N* × *N* independently controlled actuators. The total number of the required control inputs is only 2*N* (*N* inputs for selecting actuators in rows, *N* inputs for voltage control), which is greatly smaller than the total number of the actuators when *N* is large (Materials and Methods, “Control circuits for the ciliated epidermis actuation” section). The capability of this method is demonstrated in Fig. 6B and movie S5, where a ciliated matrix with 100 independently controlled actuators is developed. It can dynamically display different letters at different locations, which requires simultaneously controlling the voltages of multiple output channels.

In nature, the metachronal coordination does not always appear on stationary substrates. One typical example is the lobate ctenophore that can actively deform its appendages, where the ctene plates attach, to transit between different swimming and feeding modes (*36*). To parallel or even surpass the flow manipulation performances of their biological counterparts, soft underwater robots also need to achieve such metachronal coordination on their dynamically deforming exterior surfaces. The bendability and stretchability of the ciliated epidermis make it possible to be deployed on such surfaces. A disk-shaped ciliated epidermis with eight actuator arrays is patched to a pneumatic actuator that can expand or shrink when inflated or deflated (Fig. 6C and movie S6; Materials and Methods, “Experimental setups for dynamically deforming surface testing” section). Each actuator is 1.5 mm in length and 0.35 mm in width, and the initial space along and between the actuator arrays is 1 and 1.5 mm, respectively. The ciliated epidermis can still function even when the substrate is stretched to reach a surface area that is 1.3 times its original value. We also notice that the fluid transportation capability slightly decreases when the substrate is in the stretched state than in the compressed state, as visualized by the color maps of *U_x_* (Fig. 6C). The change in fluid transportation capability is due to the change of the interarray distance when the substrate is stretched or compressed.

Soft robots can use their soft-bodied deformation for versatile flow control, which is critical to realizing different purposes such as transportation, mixing, and multimodal locomotion (*15*, *37*–*39*). By altering the flow conditions near the dynamically deforming surfaces, the ciliated epidermis can potentially improve the interaction between the soft robots and the surrounding fluids for robot performance enhancement. For demonstration, we deploy a ciliated epidermis that is 17.5 mm by 12 mm in size on a dynamically bendable membrane driven by the external magnetic field (Fig. 6D; Materials and Methods, “Experimental setups for dynamically deforming surface testing” section). The ciliated epidermis has eight actuator arrays. Each actuator is 1 mm in length and 0.3 mm in width. The distances between adjacent actuator arrays and the adjacent actuators on the same array are 2 and 0.5 mm, respectively. The substrate is made of magnetic soft composite material and can flap back and forth with a frequency of 0.2 Hz under an oscillating magnetic field. When actuators are turned on, they all beat with mode 1 and form a metachronal wave (φ = 2π/7) transmitting from the free end to the fixed end of the substrate, boosting the transportation of the fluids along the substrate. As a result, the flow strengths along the +*y* direction are notably stronger at all instants in comparison with the flows produced when all the actuators are at rest. This result suggests that the metachronal coordination of the small actuators distributed on the surface of a flexible flapping foil may potentially help it boost the propulsion force (*40*) as more fluids can be directed downstream.

Swimmers adopting jellyfish-inspired swimming modes can capture small floating objects in the swimming direction (*38*, *41*). Enhancing the fluid transportation along the outer surface of the swimmer may help it better transport objects downstream, facilitating it to capture more objects. The ciliated epidermis can be applied to achieve this goal. For demonstration, we reduce the size of the magnetically bendable membrane to a surface area of 7 mm by 11 mm (Fig. 6E and movie S7; Materials and Methods, “Experimental setups for dynamically deforming surface testing” section). The ciliated epidermis on the membrane has three actuator arrays. Each actuator is 1.5 mm in length and 0.3 mm in width. The distances between adjacent actuator arrays and the adjacent actuators on the same array are 2 and 0.5 mm, respectively. The bendable membrane flaps with a frequency of 0.65 Hz and translates vertically with a constant speed of 2 mm/s. Before the experiments, 10 ml of fluorescein dye is injected on top of the membrane to visualize how much fluids are transported downstream. In the case when all actuators beat with mode 1 and transmit a metachronal wave (φ = 2π/7) from the free end to the fixed end of the substrate, the dye trace is wider than the case where all actuators are turned off, which indicates that the dye sheds from the top surface more rapidly and spreads to a larger area downstream with the beating of the ciliated actuators. This observation is further confirmed by a simulation result (fig. S9).

## DISCUSSION

The soft-robotic ciliated epidermises demonstrate their capability to transport fluids on various surfaces, including static and dynamically deforming 3D surfaces, which is superior to previous cilia-inspired transportation techniques that either require rigid substrates (*11*, *21*, *22*) or have a strong coupling between the actuation signals and the surface morphology (*15*, *17*). The ability to achieve the reconfigurable coordination of the multiple independently controlled actuators is another advantage over many previous ciliary devices and is the key to realizing the reconfigurable and agile control of the flow direction and strength. In addition to inducing fluid transportation in one direction, which is the only focus of many previous artificial ciliary systems (*15*, *19*), correct coordination between the actuators can also produce fluid transportation in the directions orthogonal to the actuators’ beating direction. Although a single actuator has a small motion and can only influence the flow field locally, the collective beating of many actuators together can substantially influence the global flow field around an actively deforming substrate with a much larger motion amplitude. Although the ciliated epidermises reported here are tested in 0.1 M NaDBS solution, they could also function in other liquid environments, such as in seawater, 1× phosphate-buffered saline (PBS) solution, and cerebrospinal fluid, as long as small mobile cations exist, as demonstrated in fig. S10 and a previous study (*42*). Since different electrolytes have different ion species and concentrations, the actuator performance varies with different electrolytes. The geometric designs and control signals of the actuators have to be optimized in different environmental settings.

In the future, efforts should be made to increase the maximum actuation frequency of the actuators for a better fluid manipulation performance. Except for further fine-tuning the thickness of each layer of the actuator, microstructuring the PPy film is a promising alternative. Creating microholes on the PPy surface through a surface templating technique has been used to increase the strain rate of the PPy actuator (*43*). In another study, nanochannels with hydrophobic interior surfaces have been demonstrated to greatly increase the liquid transportation speed (*44*). Such nanochannels have the potential to boost the ion diffusion rate into the polymer matrix, thus increasing the actuator’s beating frequency. The relation between the fluid manipulation performances of the ciliated epidermis and the substrate geometry should also be systematically investigated as we do find that the local curvature of the substrate influences the local flow strength and direction and the overall flow field (Fig. 4B). In addition, how to coordinate the actuators’ beating with the surface motion for flow control is another interesting topic. In the current study, we take no account of the phase shift between the metachronal wave and the substrate motion and only study the flow induced by the unidirectional metachronal wave on dynamically bending and expanding surfaces (Fig. 6). How can other types of actuators’ coordinations influence the overall flow fields of other types of dynamically deforming substrates is an open question.

The current ciliated epidermises have several limitations. First, the actuators show a short lifetime (fig. S11) due to the delamination and degradation of the PPy layer (*27*). Potential underdevelopment solutions in the literature to this challenging unsolved problem include enhancing the polymer/electrode adhesion by surface roughening (*24*) or introducing an adhesion layer (*45*), reducing the actuation potential range (*46*), synthesizing PPy composites (*47*, *48*), and engineering the micro/nanostructures of the PPy (*49*). Other bending actuators built upon inorganic materials, such as carbon nanotube or graphene (*50*, *51*), could also be explored to create durable soft-robotic ciliated epidermises. Second, the biocompatibility of the ciliated epidermis materials needs to be tested and validated for future specific biomedical applications. We envision that with the improvement to the actuators’ lifetime, the ciliated epidermis can be used as an implantable device to replace the malfunctioning cilia inside the human body where locally heterogeneous flow conditions are favored (*52*), or as a fluid flow agitation device to mimic the function of the epithelial cilia in the oviduct to increase the development rate and reduce the abnormal rate in the in vitro embryo culture where dynamically changing flow conditions are desired (*53*, *54*). Moreover, they could also be deployed on the surfaces of various underwater soft robots to augment their functional interaction with the fluidic environments for advanced locomotion and functionalities.

## MATERIALS AND METHODS

### Fabrication of the ciliated epidermis

The fabrication route for a typical soft-robotic ciliated epidermis is illustrated in Fig. 1D. In step (i), the surface of the silicon wafer is treated using a plasma cleaner for 45 s with a power of 15 W and then coated with trichloro(1*H*,1*H*,2*H*,2*H*-perfluorooctyl)silane through vacuum deposition. This step aims to weaken the adhesion between the wafer and the parylene layer for the ease of releasing the samples from the substrate in step (vi). A thin layer of parylene C film with a thickness of 0.8 μm is deposited on the wafer (PDS 2010, Specialty Coating Systems Inc.). In step (ii), the parylene C film coated on the wafer surface is roughened by iron powders for the purpose of increasing the adhesion between the parylene C and the subsequent Cr/Au layer. Specifically, the samples are first put in a petri dish. Then, the iron powders with size smaller than 450 μm are filled into the petri dish to bury the samples. Next, the petri dish is manually shaken for 6 min to produce a roughened parylene film while keeping it intact. The samples are finally cleaned with DI water and isopropanol (IPA) for the subsequent fabrication steps. In step (iii), a layer of 10-nm chromium, which is used as the adhesion layer, and a layer of 300-nm gold are sequentially deposited onto the parylene C surface. The rough parylene C surface results in a rough gold surface with *R*_a_ = 47 nm, where *R*_a_ is the arithmetic average of roughness (fig. S1). A rough gold surface can help to improve the adhesion between the gold and PPy layers (*24*). After cleaning the gold surface with DI water and IPA, the sample is ready to be used in step (iv), where a 1.3-μm-thick PPy film is deposited. The electrolyte used in the electrodeposition process is the aqueous solution of 0.1 M pyrrole and 0.1 M NaDBS. The preparation of the solution follows the recipe presented in (*26*). The thickness of the PPy film is proportional to the charge consumed per unit area (*26*). In our study, the desired PPy film thickness was realized by controlling the electrodeposition time while keeping the current constant. In step (v), polyvinyl chloride electrical tapes are first patterned on the sample surface. Then, the shape of the ciliated arrays is cut out by a laser cutter. In step (vi), the ciliated actuator arrays are peeled away from the wafer with a Scotch tape. The Scotch tape sticks to the extra part of the ciliated array. In step (vii), after the peeling-off operation, the Scotch tape and the extra part of the ciliated array are removed with scissors or the laser cutter. In step (viii), the ciliated arrays are planted onto a soft substrate to produce the soft-robotic ciliated epidermis. The soft substrates are cast with silicone elastomers such as Dragon Skin 30 (Smooth-On Inc.) or polydimethylsiloxane (SYLGARD 184, Dow Inc.). On top of the substrates are sockets that can fit the dimensions of the ciliated arrays. The ciliated arrays are fixed to the substrates using uncured elastomers the same as the substrate material. Copper wires with diameters of 50 and 30 μm are used to connect the gold electrodes to the power source. The connection between the copper wires and the electrodes is realized by silver conductive paint (SCP03B, Electrolube).

There are two methods to separate the electrodes of the actuators to produce phase shifts along the direction of the actuator array. In the first method, a multirow design is adopted (fig. S2A, i). The vias on the tape layer are still created at one end of the actuator array, and the actuator on each row is exclusively connected to a via through the serpentine connection. To release the multirow array from the substrate as a whole (step vi of Fig. 1D), the adjacent rows are still connected. To cut off the electricity links between the adjacent rows, the laser cutter is used to cut off the connection of the Cr/Au layer (fig. S2A, ii). Note that the power of the laser is fine-tuned to only cut off the parylene C and Cr/Au layers while still keeping the tape layer intact. This method is used to build the ciliated epidermis in Fig. 5. In the second method, the Cr/Au layer is exposed through slight modifications to the typical fabrication steps shown in Fig. 1D. In step (iii), the Cr layer is deposited first. Then, the parylene C films at the locations where the Cr/Au layers are supposed to be exposed are stripped from the wafer (fig. S2B, i). After that, the Au layer is deposited on top (fig. S2B, ii). The actuator array obtained by this modification at step (vii) is shown in fig. S2B (iii), where the Cr/Au layer is exposed at the root of the actuator array. By cutting through the dashed lines in fig. S2B (iii), the electrodes of the actuators can be separated. Note that the actuators can still be physically connected through the tape layer after cutting using a laser cutter. This method is used to build the actuator matrix in Fig. 6B.

### Calculation of the average flow along a line and the planar flow rate *Q*

The acquisition rate of the PIV images in this study is 500 Hz, which can help us achieve time-resolved flow field measurements. The average flow along a line $\bar{U}$ is calculated simply by $\bar{U}=\frac{\int_{l}\mathrm{vdl}}{L}$. Here, *v* is the velocity component normal or parallel to the line *l*, and *L* is the length of the line. The amount of fluids being transported per beating cycle across a specific cut line is estimated under the assumption of 2D flow. It is defined by $Q=\frac{\int_{0}^{\mathrm{nT}}\int_{l}v_{⊥}\;\text{dldt}}{\mathrm{nT}}$. Here, the velocity component *v*_⊥_ that is normal to the cut line *l* is integrated along the line within the time period *nT* that is always integer numbers of the beating period *T*. It, therefore, has a unit of square millimeters per second, corresponding to the volumetric flow rate, cubic millimeters per second, in 3D.

### Virtual particle tracing based on PIV experiments

The virtual massless particle tracing assumes that the flow fields around the actuators are 2D, and the particle velocity equals the flow velocity at the particle position. The flow fields used for particle tracing are obtained from the PIV experiments. To get the flow velocity at any location within the observation region, cubic interpolation is used to interpolate the 2D velocity-gridded data obtained from the PIV experiment. The center of mass of the particles is calculated by $X_{\text{COM}}=\frac{\sum_{i=1}^{M}X_{i}}{M}$. Here, *M* is the total number of the particles and *X_i_* is the coordinate of the *i*th particle.

### Control circuits for the ciliated epidermis actuation

The control signals for the ciliated epidermis with independently controlled DOF less than 12 are produced by three voltage output modules (NI-9269, National Instruments Corp.), each of which has four isolated output channels. Each individually controlled actuator array is exclusively connected to an output channel. However, such hardware configuration is no longer applicable if a large number of actuators have to be independently accessed, such as the 10 × 10 actuator matrix in Fig. 6B.

The coordination control of a large number of actuators is realized by a sequential scanning method. In this method, each actuator is powered by a control unit shown in fig. S12A. The voltage output, *V_o_*, of each unit is controlled by the capacitor, *C*_1_. The voltage control line *V_i_*, which connects to the output of a digital-to-analog converter (DAC), provides the power source to charge *C*_1_ and controls the voltage value *V_o_*. A solid-state relay (SSR), which is enabled by the enable line, *En*, is used as a switch to enable or disable the charging of the capacitor. For an *N* × *N* actuator matrix, *N* × *N* control units are needed so that each actuator has an exclusive control unit. To control these units, *N* voltage control lines and *N* enable lines are required. Specifically, each column of the units is attached to the same voltage control line, and each row of the units is attached to the same enable line.

The sequential scanning method is illustrated in fig. S12B. For ease of illustration, we only consider the control sequence for producing a transmitting wave along the diagonal direction of a 3 × 3 actuator array. The time-varying coordination of the actuators is discretized in time to produce a sequence of frames describing the patterns of the output voltages of the actuator matrix. It takes multiple steps to achieve one frame, i.e., each pattern is updated row by row. For an *N* × *N* actuator matrix, it takes *N* steps to update one frame. This scanning strategy requires that the control units that have already been scanned can keep the voltages (or the voltages do not drop rapidly) when the circuit is updating the voltages of other control units. This requirement can be realized by using a capacitor with a large capacitance (100 μF) in each control unit. The updating speed of each frame is confined by the response time of the SSRs and the matrix size *N*. In movie S5, we notice that the actuators that are deactivated still shake slightly. This may possibly be caused by two reasons. First, since the grounds of all actuators are connected together, the current flowing through the ground can pull the ground potential up relative to the reference electrode, changing the redox state of the actuators that are not activated. Second, since all the electrodes are immersed in the same electrolyte, weak currents may flow between the adjacent electrodes, causing the weak electricity coupling between the actuators. One solution to this problem is to increase the scanning rate of the circuit. The updating time of the control circuit, *T*_update_, can be calculated by the following equation

$$T_{\text{update}}=N_{\text{row}}(N_{\text{column}}T_{\text{DAC}}+T_{\text{SSR}–\text{open}}+T_{\text{charging}}+T_{\text{SSR}–\text{close}})$$

*N*_row_ and *N*_column_ are the numbers of the rows and columns of the control units being activated. *T*_DAC_ ≈ 10.4 ms is the time required to communicate with the DAC (MCP4728, Microchip Technology Inc.). *T*_SSR−open_ and *T*_SSR−close_ are the time required to open and close the SSR (AQV212SX, Panasonic Electronic Components), which are 2 and 0.2 ms, respectively. *T*_charging_ is the charging time of the capacitor. For a 100-μF capacitor connected with a 100-ohm resistance in series, it takes around 3 ms to reach 90% of its maximum charge capacity. The updating time of a 10 × 10 control unit matrix is around 1092 ms. There is still a huge room for improving the updating time of the circuit. In the current design, the communication protocol between the microcontroller and the DAC chip is I2C. Replacing DAC chips with ones supporting faster communication protocols, such as SPI, can decrease *T*_DAC_. In addition, the microcontroller we use in this work is ATMEGA328P-PU, which can only communicate with one I2C device every time. Replacing it with other microcontrollers supporting parallel communication can greatly reduce *T*_update_ by decreasing the multiplier of *T*_DAC_.

### Experimental setups for dynamically deforming surface testing

For experiments on the dynamically stretchable surfaces, the ciliated epidermis is deployed on a pneumatic soft actuator, as shown in fig. S13A. The actuator is inflated or deflated by a syringe pump through a tube. The soft actuator is made of Dragon Skin 0030. For experiments on the dynamically bendable substrate, the ciliate epidermis is deployed on a membrane made of magnetic soft composite material, as shown in fig. S13B. The membrane is magnetized longitudinally. During the experiments, one end of the membrane is fixed to a fixture, and its flapping motion is controlled by a permanent magnet underneath.

### Simulations on the 2D flow fields

The 2D fluid flow simulation here aims to answer the questions in the form of how the flow field is influenced if the actuators are fabricated and controlled in the desired way. Therefore, the deformations of the actuators are fully prescribed and are not influenced by the hydrodynamic loads in simulation. Assuming that each layer of the actuator is homogeneous in thickness and material properties, the actuator should be able to bend, maintaining a constant curvature (*55*). The deformation of the actuator, under the assumptions of classic beam theory, is related to the curvature by ε*_xx_* = −κ*y*. Here, ε*_xx_* is the longitudinal strain, κ is the curvature of the neutral axis, and *y* is the coordinate in the transverse direction. Given the time-varying κ, the strain of the actuator at each instant of time can be determined. The flow field around the actuator is solved by numerically solving the Navier–Stokes (N-S) equation. The simulation is conducted in commercial software COMSOL 6.0.

To investigate how the actuator length and the planting angle influence the flow filed around the actuators, the N-S equation is solved within the domain with the boundary conditions shown in fig. S6A. The time-averaged flow velocities are calculated along the red dashed line in fig. S6A. The red dashed line is placed in a position that it just cuts the actuator tip when the *y* coordinate of the actuator tip reaches the maximum. Its length is two times the actuator’s length. To investigate how the ciliated epidermis helps the spreading of the dye on a dynamically deforming membrane, virtual particles are constantly released from the upper surface of the membrane with an equal time interval (0.1 s) when the membrane is flapping (fig. S9B). The virtual particles have a radius of 0.1 μm and therefore are assumed to move following the Maxey-Riley equation (*56*). To mimic the experimental conditions in Fig. 6E, the simulation domain has a uniform background flow downward. The fluid transportation of the ciliated epidermis is modeled by applying a moving wall boundary condition on the top surface of the membrane. The final positions and the trajectories of the released particles are shown in fig. S9 (D and E).

### Statistics

In the figures, the error bars represent the standard error of the mean. The number of the trials for each plot can be found in the corresponding figure caption.
