# Supplementary material for: Soft-robotic ciliated epidermis for reconfigurable coordinated fluid manipulation
Source: Sci Adv. 2022 Aug 26;8(34):eabq2345. doi: 10.1126/sciadv.abq2345 (PMC9417179; doi:10.1126/sciadv.abq2345)
Supplement: Supplementary file 1 — Figs. S1 to S13 [file sciadv.abq2345_sm.pdf]

Science Advances
AAAS

Supplementary Materials for

Soft-robotic ciliated epidermis for reconfigurable coordinated fluid manipulation

Ziyu Ren *et al.*

Corresponding author: Wenqi Hu, wenqi@is.mpg.de; Metin Sitti, sitti@is.mpg.de

Sci. Adv. **8**, eabq2345 (2022)
DOI: 10.1126/sciadv.abq2345

The PDF file includes:

Figs. S1 to S13
Legends for movies S1 to S7

Other Supplementary Material for this manuscript includes the following:

Movies S1 to S7

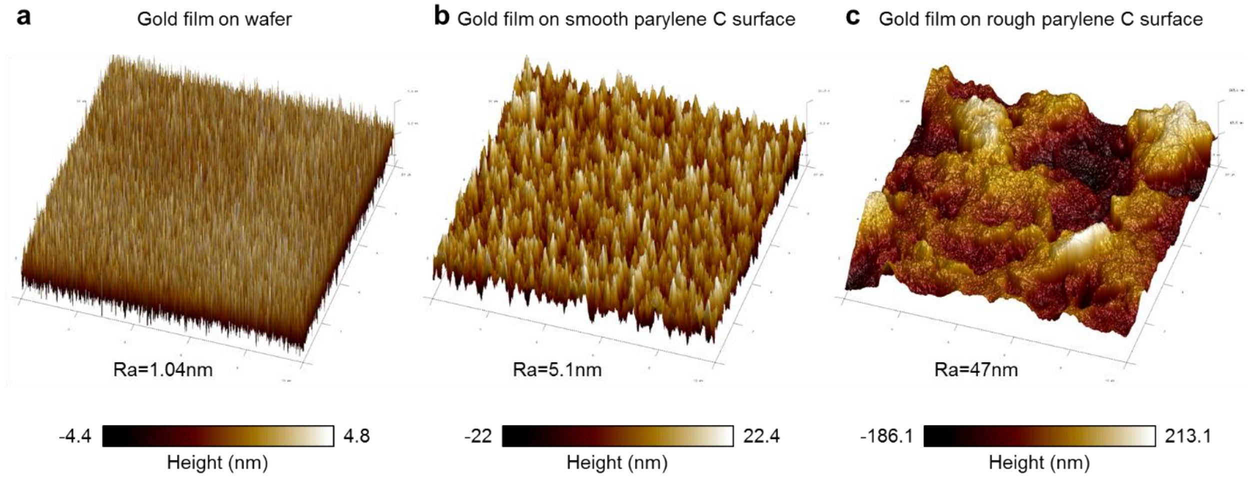

Figure S1. Atomic force microscope-based 3D surface roughness measurements of the gold layer. (a) The gold film deposited on the wafer. (b) The gold film deposited on the prime parylene C surface. (c) The gold film deposited on the parylene C surface being roughened. A rough gold surface can be obtained by roughening the parylene C layer underneath. R_a is the arithmetic average of roughness.

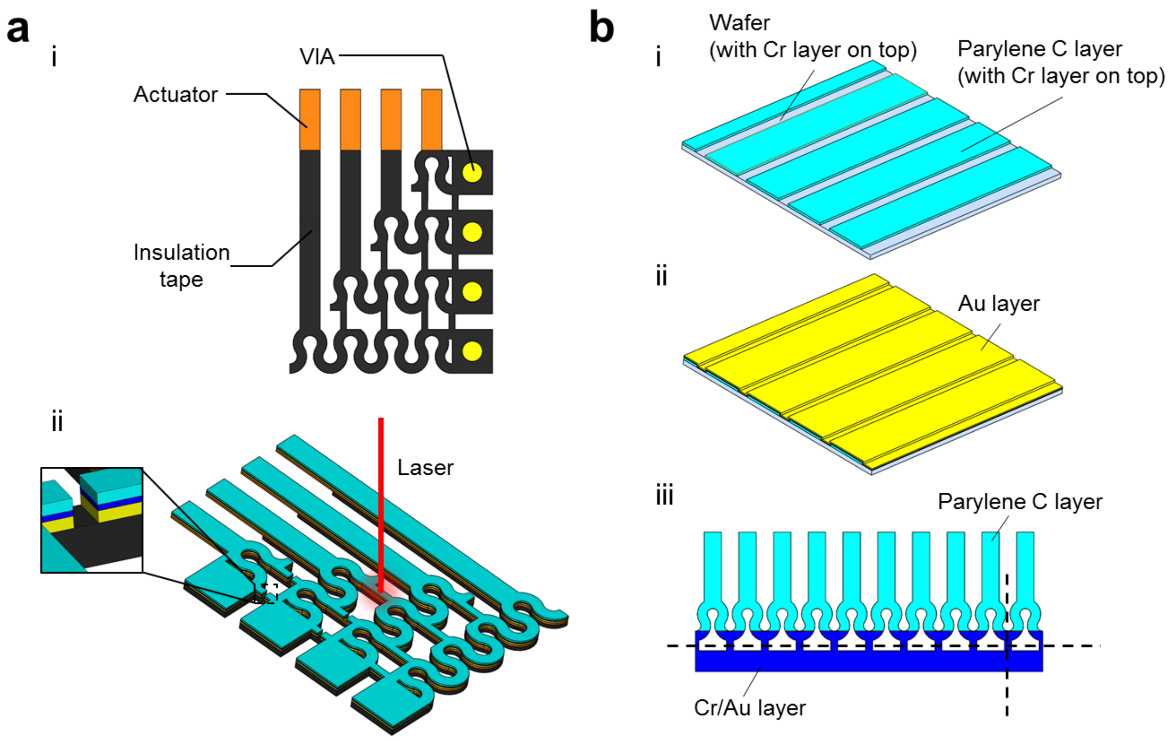

Figure S2. Two strategies to fabricate independently controllable actuators on the same actuator array. (a) Multi-row design for independent control of the actuators on the same array. The gold layer is exposed at one end of the array (i). The electricity connections between the rows are cut off by a laser cutter (ii). (b) Exposing the gold layer at the root of the actuator. A layer of Cr is first deposited on top of the parylene C layer. The parylene C layer where the gold layer needs to be exposed is stripped (i). The Au layer is then deposited on top (ii). The actuator electrodes are finally separated by cutting through the black dashed lines (iii).

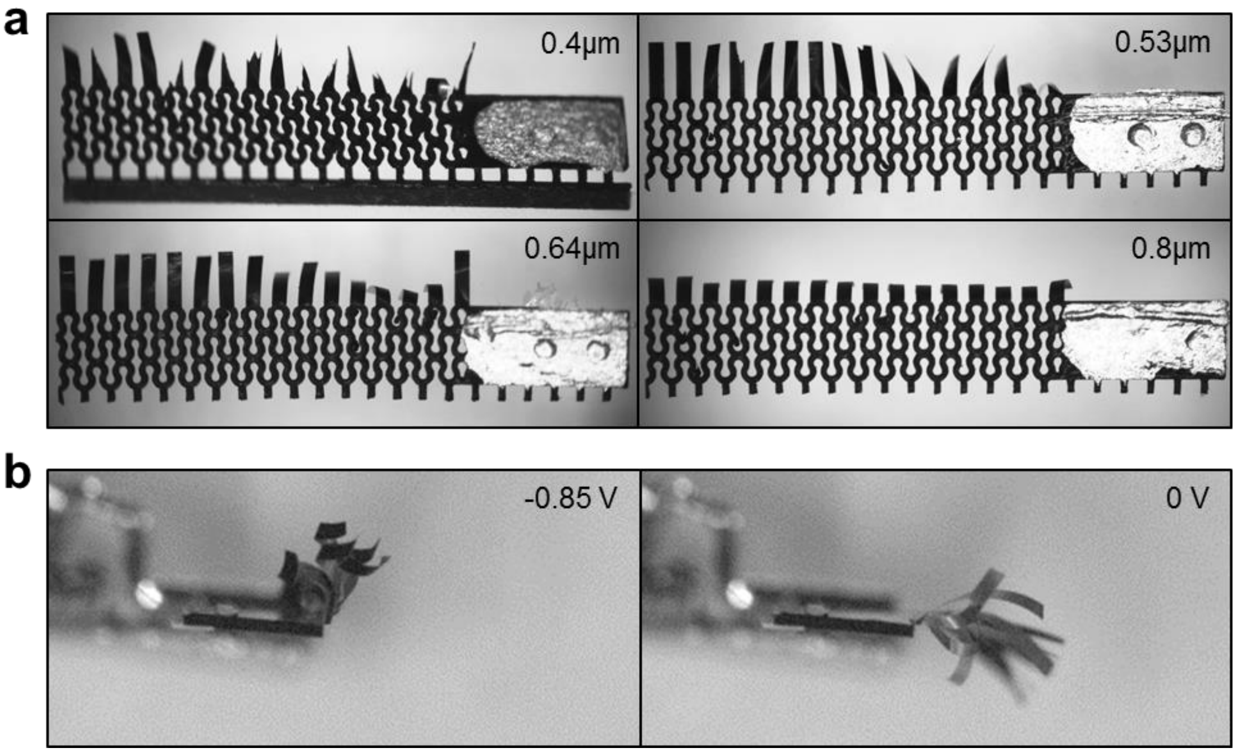

Figure S3. The influence of different geometric design parameters on the actuator quality. (a) The influence of the parylene C layer thickness on the quality of the free-standing actuators. The actuator array just released from the wafer is intact and homogeneous only if the thickness of the parylene C layer reaches 0.8 μm. (b) The influence of the actuator length. Long actuators (2.5 mm) are easy to get entangled during actuation.

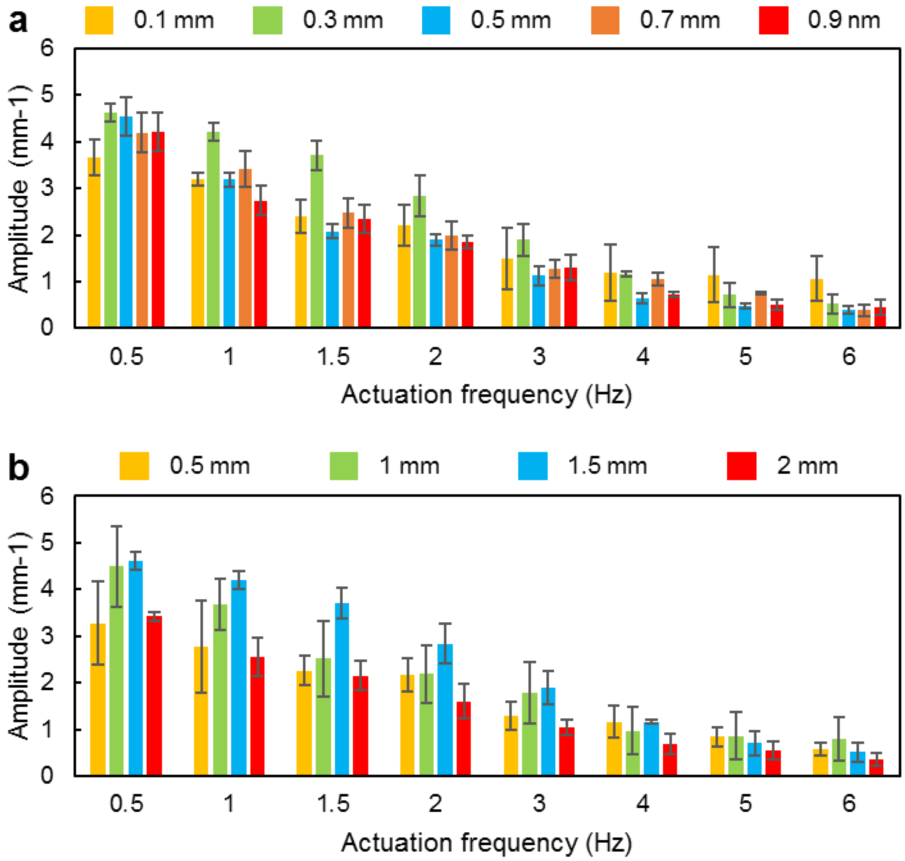

Figure S4. The influence of other geometric design parameters on the bending amplitude. (a) The influence of the width. (b) The influence of the length. The values are averaged from the measurements of three samples. The error bars represent the standard error of the mean.

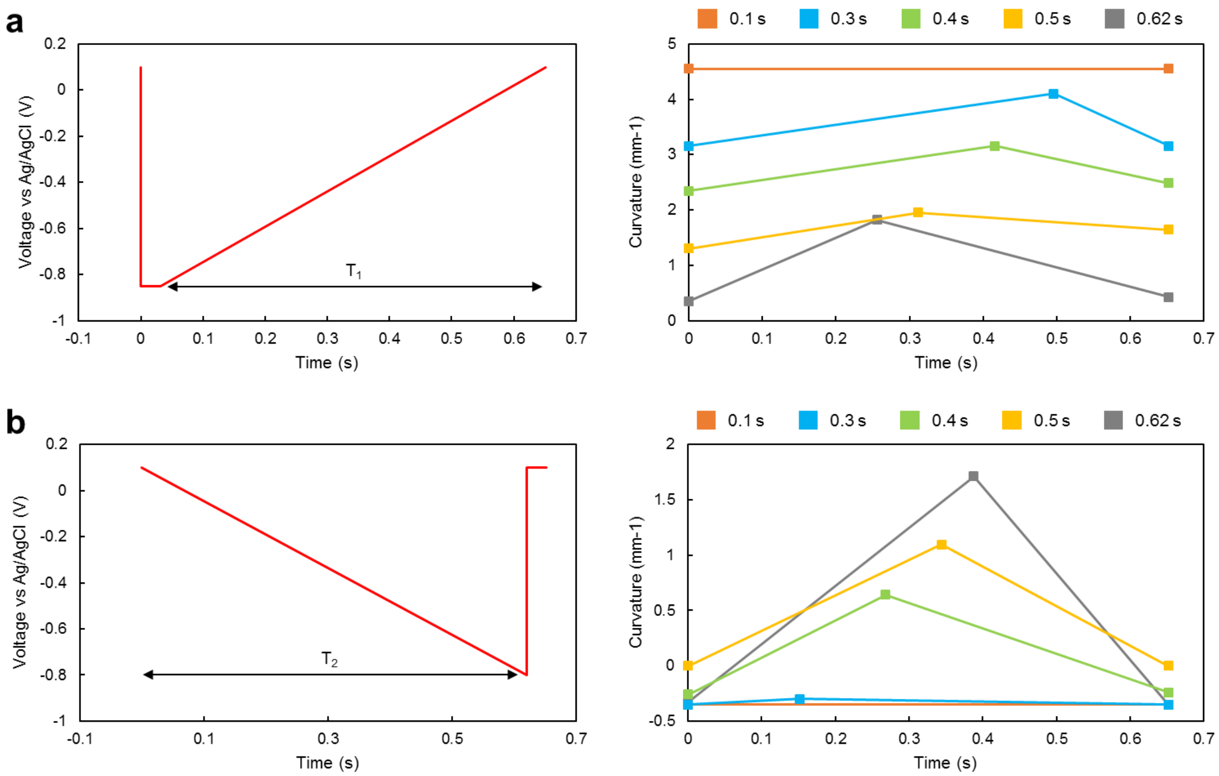

Figure S5. The influence of the control signal waveform on the beating performance of the actuator. In (a) and (b), the durations of oxidation and reduction vary with T_1 and T_2. The bending curvatures of the actuators are measured when they are fully reduced or fully oxidized. When T_1=0.62 s and T_2=0.62 s, the actuators can achieve the largest bending amplitude.

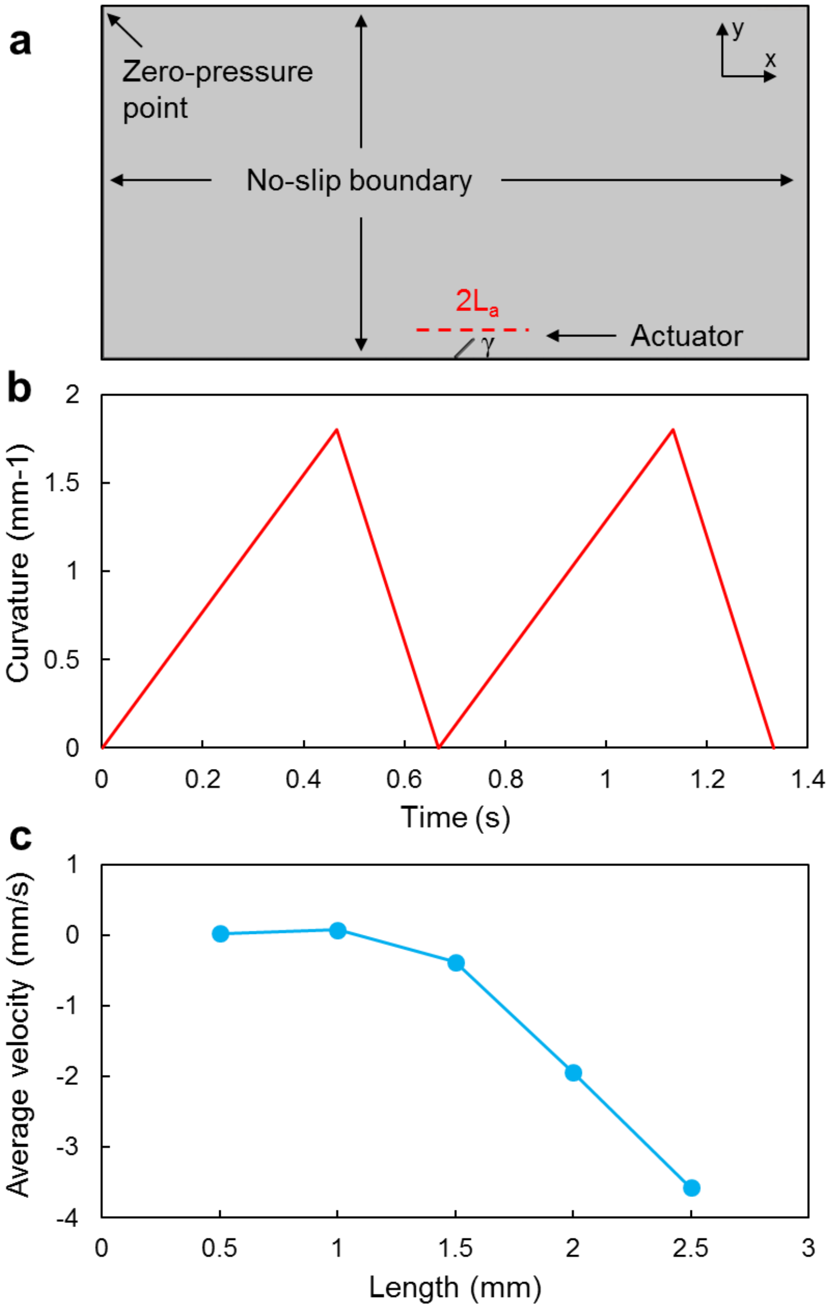

Figure S6. Simulation about the effect of the actuator length on the fluid transportation performance. (a) The simulation domain and the boundary conditions. γ=45° is the planting angle. (b) The curvature variation prescribed to the actuator. (c) The variation of the average velocity with the actuator length. The time-averaged velocities are calculated along the red dashed line shown in (a). The red dashed line is placed in a position where it just cuts the actuator tip when the y coordinate of the actuator tip reaches the maximum. The red dashed line is $2L_a$ (L_a is the actuator length) in length.

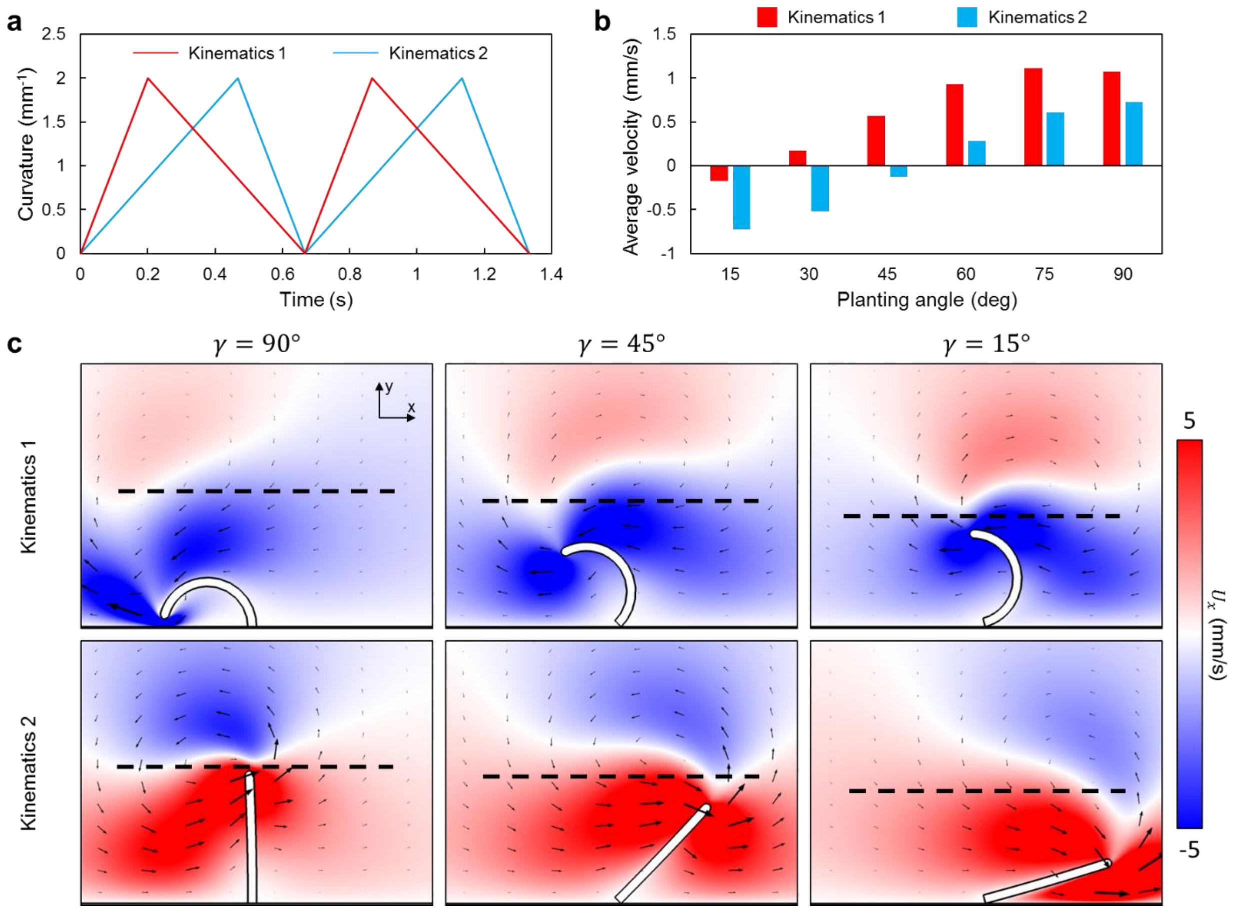

Figure S7. Simulation about the effect of the planting angle on the fluid transportation performance. (a) The curvature variation prescribed to the actuator. The actuator is 1.5 mm in length. (b) The time-averaged velocities obtained at different planting angles and beating kinematics. The time-averaged velocity is calculated along the black dashed line shown in (c). The black dashed line is placed in a position where it just cuts the actuator tip when the y coordinate of the actuator tip reaches the maximum. The black dashed line is 3 mm in length. (c) The instantaneous flow fields obtained when the actuators achieve the largest and the minimum bending curvatures. When $\gamma=90^{\circ}$, the actuator has the minimum blockage to fluids at the largest curvature while has the maximum blockage to fluids at the minimum curvature. When $\gamma=15^{\circ}$, the actuator has the maximum blockage to fluids at the largest curvature while has the minimum blockage to fluids at the minimum curvature.

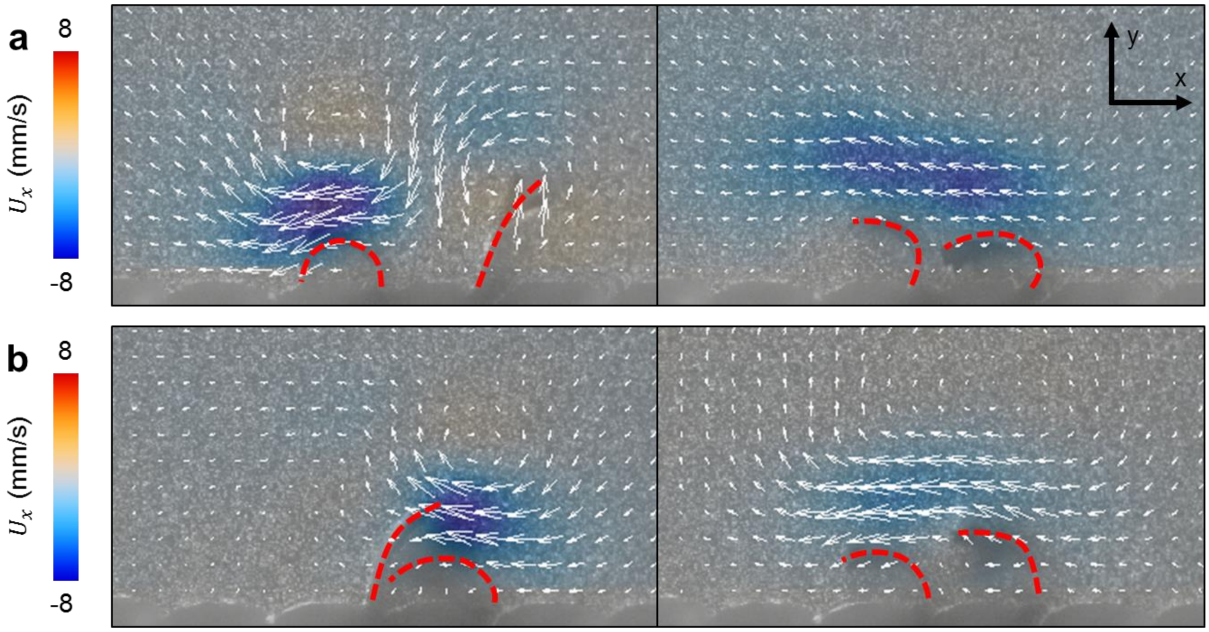

Figure S8. The influence of the phase shift between the actuator arrays. (a) The left actuator leads the beating by $\varphi_x = 2\pi/7$. (b) The right actuator leads the beating by $\varphi_x = 2\pi/7$. This improper phase shift between the actuator arrays decreases the peak flow velocity achieved by the actuator pair. The bendings of the actuators are indicated by the red dashed curves.

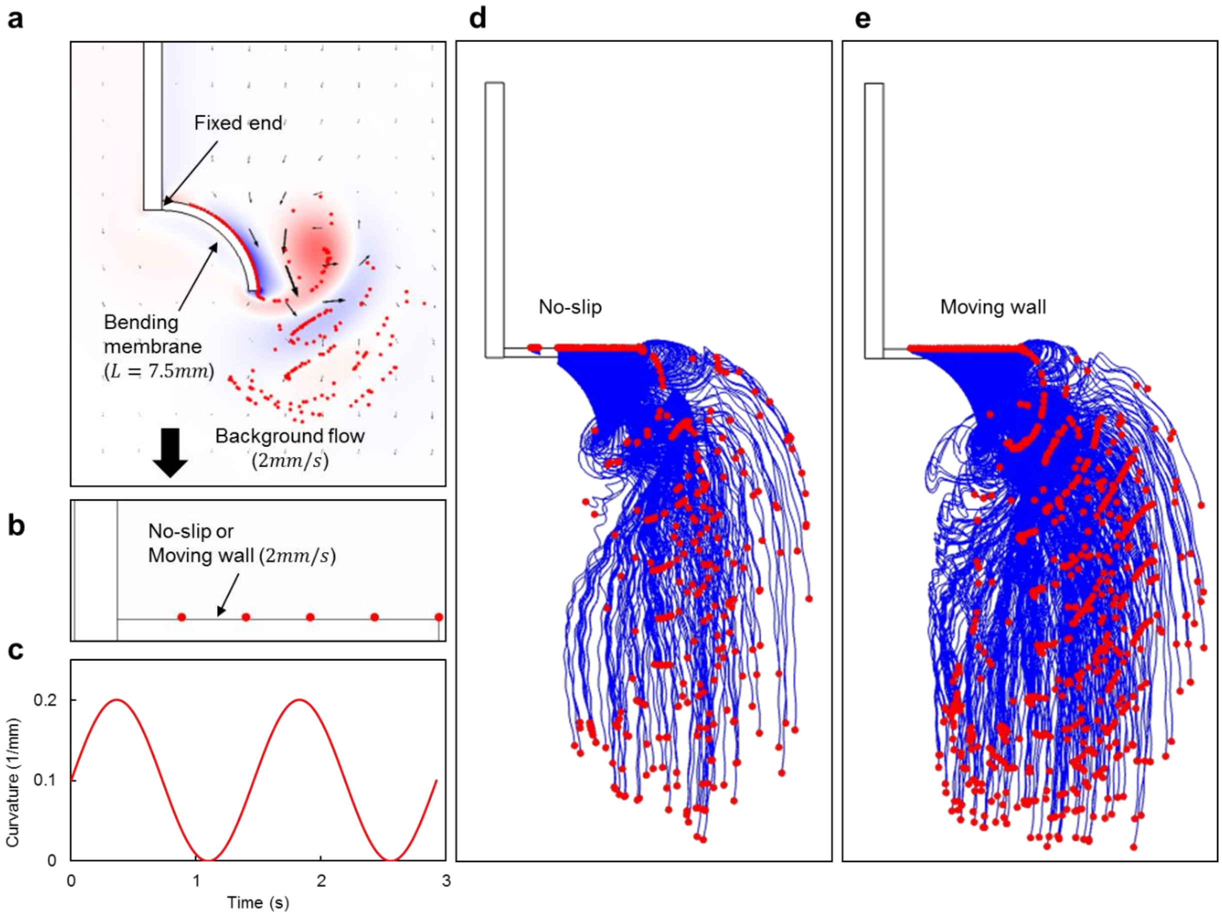

Figure S9. Influence of the near-surface fluid transport on a dynamically bendable substrate. (a) The simulation domain. (b) Small particles are released from the upper surface every 0.1 s from the locations shown in the figure. The fluid transportation induced by the ciliated epidermis near the substrate is mimicked by applying a moving wall boundary condition. (c) The curvature variation of the dynamically bendable membrane in two bending cycles. (d-e) The comparison of the particle trajectories at different boundary conditions after 10 bending cycles.

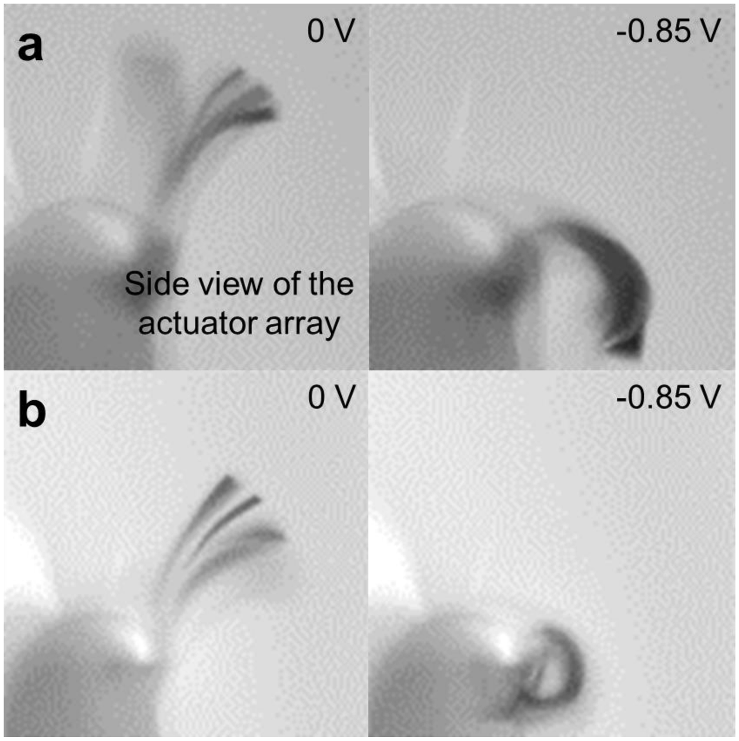

Figure S10. Actuation in other electrolytes. (a) In 1X Phosphate-Buffered Saline (PBS) solution. (b) In artificial seawater. In both (a) and (b), the actuators are driven by a control signal with a square waveform ranging from -0.85 V to 0 V at a frequency of 1.5 Hz. The photos are the side views of an actuator array.

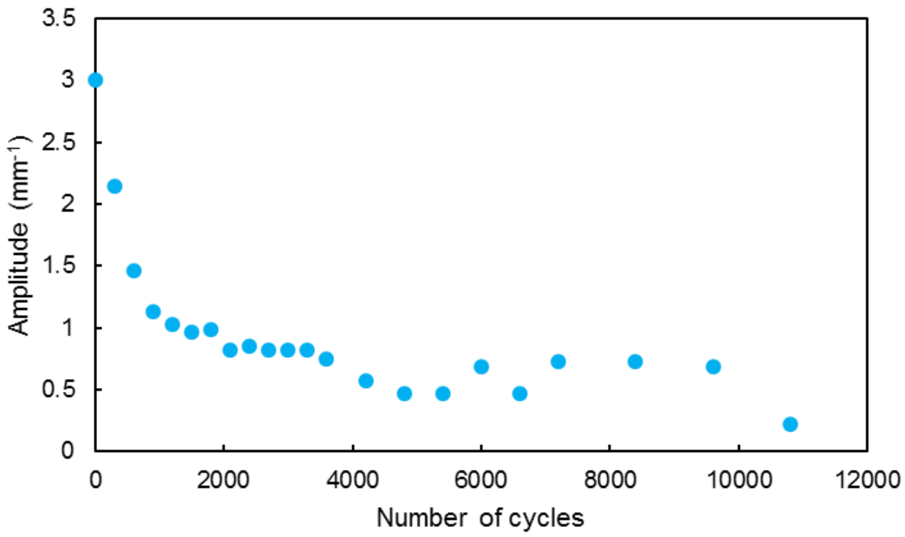

Figure S11. The variation of the beating amplitude with the actuation cycle. The actuators are driven by a squared control waveform ranging from -0.85 V to 0 V at a frequency of 1 Hz. The beating amplitude drops rapidly in the first 2000 cycles and then flattens.

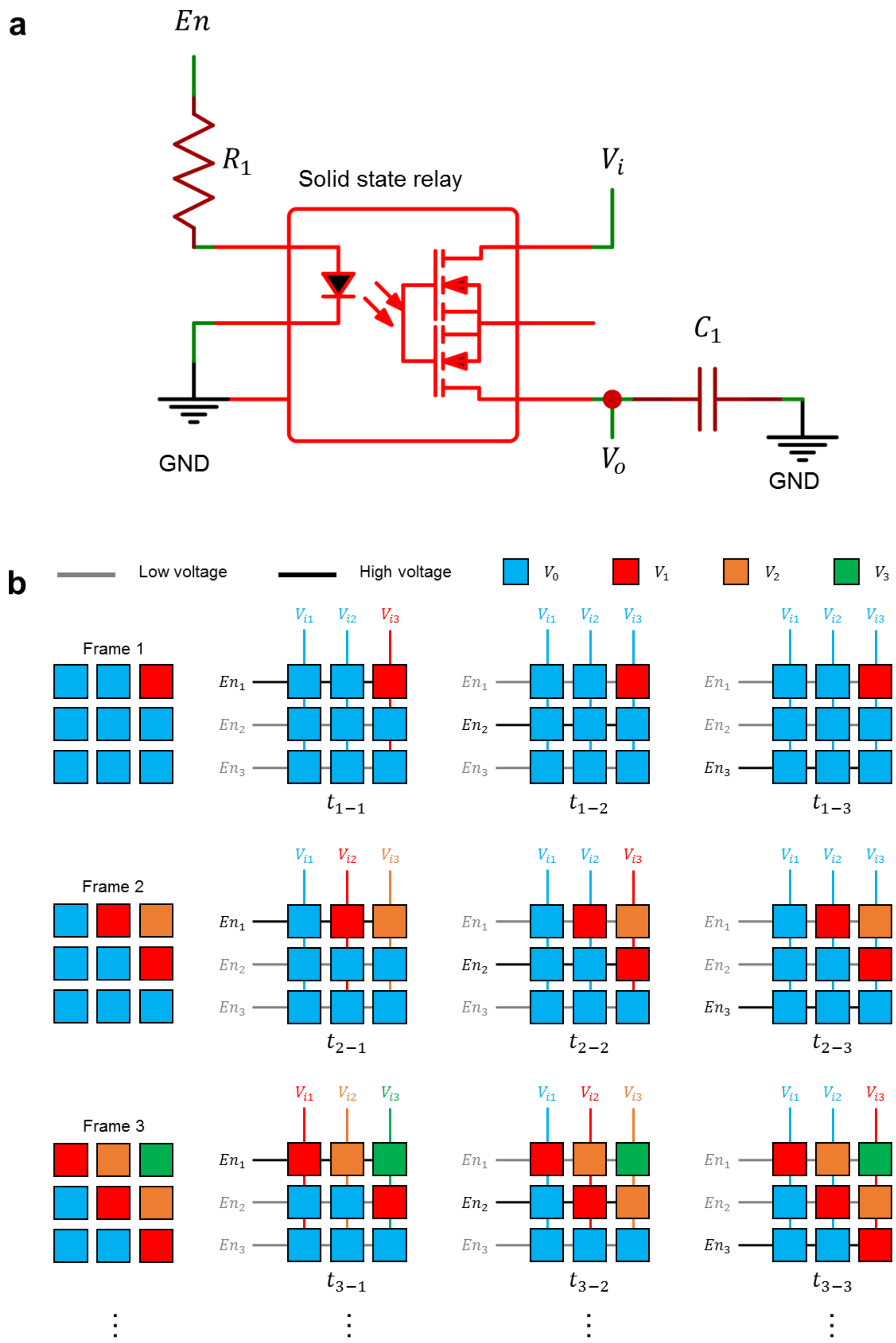

Figure S12. Circuit diagram and the sequential scanning method to generate patterns on a $N \times N$ actuator matrix. (a) The circuit design of a control unit for a single actuator. (b) Schematic illustration of the sequential scanning method to produce time-varying patterns on a 3×3 actuator matrix.

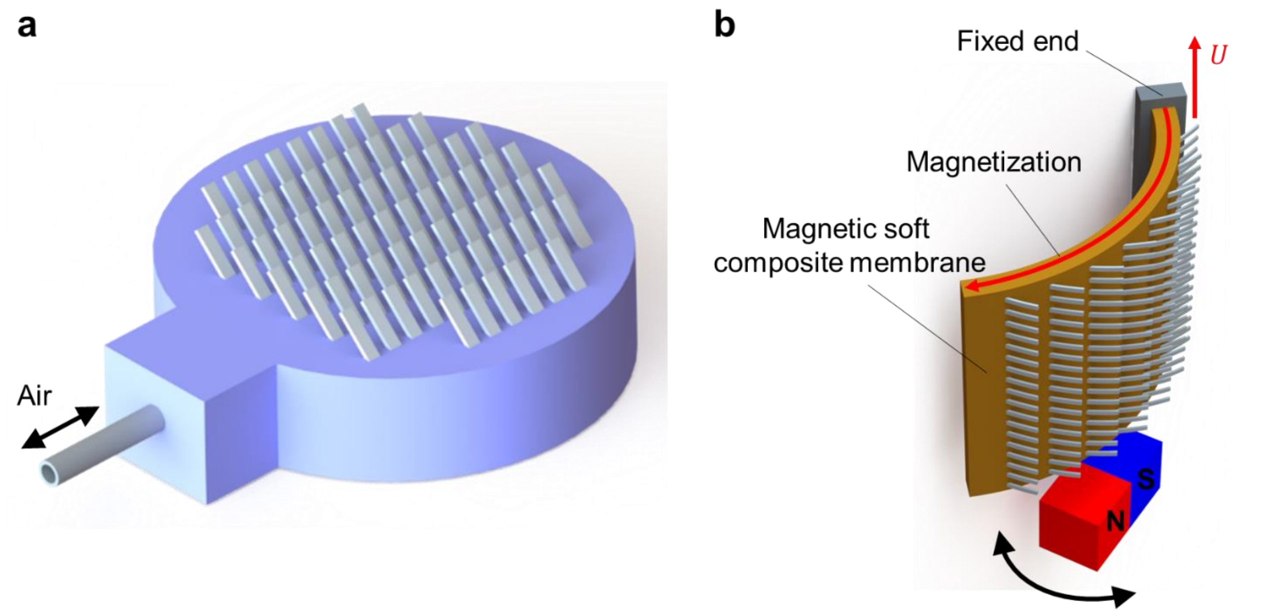

Figure S13. Experimental setup for dynamically deforming surface tests. (a) A soft pneumatic actuator used to realize the dynamically stretchable surface. (b) A magnetic soft membrane used to realize the dynamically bendable substrate. One end of the membrane is fixed to a translational stage that can provide a vertical translational speed.

Supplementary Movies

Movie S1:

Fluid manipulation performance of the ciliated epidermis on a flat substrate. The phase shifts are produced between the actuator arrays, and all actuators on the same array beat in phase. Fluid flows are produced along and perpendicular to the substrate.

Movie S2:

Fluid manipulation performance of the ciliated epidermis on a sinusoidal 3D surface. The phase shifts are produced between the actuator arrays, and all actuators on the same array beat in phase. The reversal point can be shifted along the substrate.

Movie S3:

Fluid manipulation performance of the ciliated epidermis that can produce phase shifts between the actuator arrays and along the actuator arrays.

Movie S4:

Fluorescein dyes are pumped out of the tube by the ciliated epidermis deployed inside.

Movie S5:

The 10×10 actuator matrix displays different letters at different locations using the sequential scanning method. Letters ‘M’, ‘P’, ‘I’, ‘I’, and ‘S’ are sequentially displayed.

Movie S6:

Fluid transportation on a dynamically stretchable surface.

Movie S7:

Fluid transportation on a dynamically bendable surface that translates vertically. The fluorescein dyes spread to a larger area downstream when the ciliated epidermis is switched on.
